# Hidden Diversity Hampers Conservation Efforts in a Highly Impacted Neotropical River System

**DOI:** 10.3389/fgene.2018.00271

**Published:** 2018-07-24

**Authors:** Naiara G. Sales, Stefano Mariani, Gilberto N. Salvador, Tiago C. Pessali, Daniel C. Carvalho

**Affiliations:** ^1^Ecosystems and Environment Research Centre, School of Environment & Life Sciences, University of Salford, Salford, United Kingdom; ^2^Laboratório de Ecologia e Conservação, Universidade Federal do Pará, Belém, Brazil; ^3^Museu de Ciências Naturais PUC Minas, Pontifícia Universidade Católica de Minas Gerais, Belo Horizonte, Brazil; ^4^Programa de Pós-graduação em Biologia de Vertebrados, Pontifícia Universidade Católica de Minas Gerais, Belo Horizonte, Brazil

**Keywords:** barcode, biodiversity, cryptic diversity, Doce River, ichthyofauna, molecular identification

## Abstract

Neotropical Rivers host a highly diverse ichthyofauna, but taxonomic uncertainty prevents appropriate conservation measures. The Doce River Basin (DRB), lying within two Brazilian threatened hotspots (Atlantic Forest and Brazilian Savanna) in south-east Brazil, faced the worst ever environmental accident reported for South American catchments, due to a dam collapse that spread toxic mining tailings along the course of its main river. Its ichthyofauna was known to comprise 71 native freshwater fish species, of which 13 endemic. Here, we build a DNA barcode library for the DRB ichthyofauna, using samples obtained before the 2015 mining disaster, in order to provide a more robust biodiversity record for this basin, as a baseline for future management actions. Throughout the whole DRB, we obtained a total of 306 barcodes, assigned to 69 putative species (with a mean of 4.54 barcodes per species), belonging to 45 genera, 18 families, and 5 orders. Average genetic distances within species, genus, and families were 2.59, 11.4, and 20.5%, respectively. The 69 species identified represent over 76% of the known DRB ichthyofauna, comprising 43 native (five endemic, of which three threatened by extinction), 13 already known introduced species, and 13 unknown species (such as *Characidium* sp., *Neoplecostomus* sp., and specimens identified only at the sub-family level Neoplecostominae, according to morphological identification provided by the museum collections). Over one fifth of all analyzed species (*N* = 16) had a mean intraspecific genetic divergence higher than 2%. An integrative approach, combining NND (nearest neighbor distance), BIN (barcode index number), ABGD (automatic barcode gap discovery), and bPTP (Bayesian Poisson Tree Processes model) analyses, suggested the occurrence of potential cryptic species, species complex, or historical errors in morphological identification. The evidence presented calls for a more robust, DNA-assisted cataloging of biodiversity-rich ecosystems, in order to enable effective monitoring and informed actions to preserve and restore these delicate habitats.

## Introduction

Neotropical rivers host an extremely diverse ichthyofauna, but anthropogenic impact associated with the occurrence of many still undescribed or unknown species may hamper conservation effort (Reis et al., 2016; Ely et al., 2017). Due to increasing, rapid anthropogenic environmental impacts (e.g., pollution, siltation, mining, damming), biodiversity in Neotropical rivers may be lost before scientists can fully describe and comprehend it (Agostinho et al., 2005).

Effective biodiversity conservation relies on unequivocal and precise species identification, especially in the case of ecosystems that underwent degradation and require restoration. However, high biodiversity regions, such as the neotropics, and the increasingly reduced budget for basic taxonomical research, have led to the so-called “taxonomic impediment” or “poor taxonomy”, in which the shortage of funding and trained taxonomists, and the gaps in taxonomic knowledge, have delayed advances in assessment and description of biodiversity or even contributed to overestimate or underestimate species richness due to species misidentification or taxonomic confusions (Taylor, 1983; Ely et al., 2017).

The DNA barcoding initiative offers a powerful and cost-effective tool to assist with the detection of cryptic species and flag potentially problematic taxa, with the standard universal COI marker having proven particularly successful in invertebrates (Hebert et al., 2004a), birds (Hebert et al., 2004b), and fish (Ward et al., 2005; Hubert et al., 2008; Valdez-Moreno et al., 2009; Carvalho et al., 2011; Rosso et al., 2012). For effective DNA barcode performance, intraspecific variability must be lower than variability among congeneric species, the so-called ‘Barcode Gap’ (Meyer and Paulay, 2005). While the barcode gap tends to be around <1–2% sequence variability within species in most fish, there are exceptions (Hurst and Jiggins, 2005), especially in the case of recently diverged species (Vinas and Tudela, 2009; Shum et al., 2017). Moreover, the unambiguous identification of species from early larval stage to adulthood can aid a variety of conservation management actions. Accurate molecular identification may contribute to improving management and sustainability of long term fisheries (Metcalf et al., 2007), tracking invasive species (Corin et al., 2007; Carvalho et al., 2009), offer insights into community ecology (Pfenninger et al., 2007) and genetic certification of species used in restocking programs (Metcalf et al., 2007), as well as improving fundamental knowledge on cryptic and putatively new species (Pereira et al., 2011). Furthermore, molecular identification of eggs and larvae can provide data regarding spawning and recruitment areas, supporting a definition of priority areas for conservation (Becker et al., 2015; Frantine-Silva et al., 2015).

DNA barcode libraries have been developed for several Neotropical river systems as a biodiversity identification tool, and have contributed to reveal the existence of putatively cryptic/new fish species (Carvalho et al., 2011; Pereira et al., 2011; Gomes et al., 2015; Pugedo et al., 2016; Nascimento et al., 2016). However, the biodiversity complexity remains unknown in many already impacted catchments in Brazil. One emblematic case is that of the Doce River Basin (DRB), which faced the worst environmental accident reported for any South American catchment, in the form of the largest tailings dam burst in modern history; as a result, a toxic mud (i.e., extreme high concentration of iron) spread along its main river course, affecting wild communities, as well as the local human populations (Fernandes et al., 2016; Neves et al., 2016). As the local riverine human communities rely on fisheries for their livelihood (e.g., source of income and subsistence, resource for ecotourism), understanding the impacts of this disaster on the ichthyofauna is crucial for effective management actions (Ecoplan-Lume, 2010; GFT, 2015; Neves et al., 2016). Moreover, the recovery of fish populations in DRB, after the ecological disaster, relies on the recolonization of the main course of this river and on the diversity, size, and conservation status of the remnant fish populations in the tributaries (Olds et al., 2012).

The DRB runs through two Brazilian biodiversity hotspots (Atlantic forest and Brazilian Savanna) located in south-east Brazil (Myers et al., 2000). The river is 853 km long and the catchment covers a total drainage area of 83.400 km^2^, between the states of Minas Gerais (86%) and Espírito Santo (14%), an area inhabited by three million people. DRB harbors a rich ichthyofauna, including several undescribed species, with the number of presently recognized native species summing up to 71 (Vieira, 2009). The Santo Antônio River, the second largest tributary of the Doce, was selected as a conservation priority area, since it hosts a great number of species considered endemic and threatened by extinction (Vieira et al., 2000; Vieira and Alves, 2001; Rosa and Lima, 2005). Historically, DRB is affected by human impacts by many ways. Native forest cover only 27% of DRB area (ANA, 2016), and the remained area is used to cattle, forestry, agriculture, and mining (Vieira, 2009), resulting in high rate of siltation (da Silva et al., 2011). Habitat fragmentation lead by hydroelectric construction is also affecting DRB, where there are 40 hydroelectric built along main channel of Doce River and its principal tributaries (ANEEL, 2010). However, without accurate biodiversity knowledge, species conservation may be hindered in this river system, and it had already been suggested that the environmental disaster involving the mining collapse could have led to the depletion/extinction of many still unknown endemic species (Fernandes et al., 2016). Here, we develop a DNA barcode library for the DRB ichthyofauna, using data obtained prior to the dam burst environmental disaster, contributing to an improved biodiversity baseline record for this recently impacted ecosystem.

## Materials and Methods

### Sampling

**FIGURE 1:**
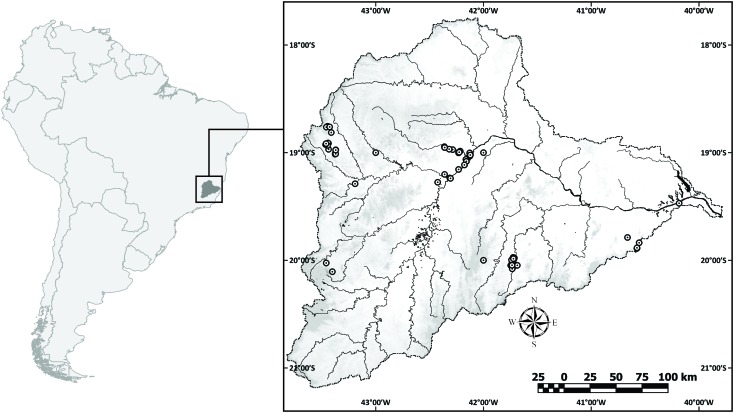
Map of Doce River Basin, including sample sites distribution.

We obtained fish tissue samples from 306 specimens collected between 2011 and 2015 along the main river channel and tributaries (**Figure 1**), identified and deposited by taxonomists in four Brazilian ichthyological collections: PUC Minas Natural History Museum (MCNIP), Museu de Biologia Professor Mello Leitão (MBML), Museu de Zoologia da Universidade Estadual de Campinas (ZUEC), and Núcleo de Pesquisas em Limnologia, Ictiologia e Aquicultura (NUPELIA). All analyzed specimens were photographed, geo-referenced, and identified to the lowest taxonomic level from identification keys or previously published works (Vari, 1992; Albert et al., 1999; Castro and Vari, 2004; Zanata and Camelier, 2009).

### Ethics Statement

All fish analyzed in this study were collected in accordance with Brazilian legislation (Collection license 6421-1, number 5498740) or obtained from Ichthyological collections. Fish were collected, and euthanized; samples of fins were clipped from each individual and stored in absolute ethanol for subsequent molecular analysis. Specimens were fixed in 10% formaldehyde and then stored in 70% ethanol.

### DNA Extraction, Amplification, and Sequencing

Genetic analyses were conducted, whenever possible, on a minimum of five specimens from different sample sites per species. DNA extraction followed the salting out protocol (adapted from Aljanabi and Martinez (1997)). The cytochrome c oxidase I (COI) gene (650 bp) was amplified by polymerase chain reaction (PCR) using the primers FishF1/FishR1 described by Ward et al. (2005) and the Cocktail COI-3/C_FishF1t1-C_FishR1t1 described by Ivanova et al. (2007), and following the PCR protocol described in Gomes et al. (2015). The PCR products were visualized on 1% agarose gel, alongside negative controls and a size ladder, and positive amplifications were selected for DNA sequencing. DNA sequencing was conducted in both directions in an automated DNA analyzer ABI 3500 (Life Technologies).

### Data Analysis

Barcode sequences were edited using DNA Baser^®^ v.3.5.4 (DNA Sequence Assembler v4 (2013), Heracle BioSoft^1^) and SeqScape v.2.1.1 (Applied Biosystems, Foster City, CA, United States) (Díaz et al., 2016) softwares. DNA alignment was conducted using the CLUSTAL W alignment tool (Thompson et al., 1997). The neighbor-joining (NJ) trees (Saitou and Nei, 1987) and genetic distances estimations, using the K2P (Kimura-2-parameter) nucleotide evolution model (Kimura, 1980) were generated using MEGA 7 software (Kumar et al., 2016).

Intra- and inter-specific genetic distances, nearest neighbor distance (NND), and the barcode gap were calculated in the on-line Barcode of Life Data System (BOLD) Workbench^2^ (Ratnasingham and Hebert, 2007). The NND was used to estimate the minimum genetic distance between pairs of species. Different approaches were used to delimitate the Molecular Operational Taxonomic Units (MOTUs), two clustering algorithms [barcode index number (BIN) and Automatic Barcode Gap Discovery (ABGD)] and one phylogenetic-coalescent method Bayesian Poisson Tree Processes model (bPTP). The BIN (Ratnasingham and Hebert, 2013) was estimated automatically in BOLD Workbench and allowed comparing DNA barcodes obtained here with other river basins that have a comprehensive DNA Barcode library, such as the São Francisco, the Mucuri, the Jequitinhonha, the Paraná, and the Paranaíba River Basins (Carvalho et al., 2011; Pereira et al., 2011; Gomes et al., 2015; Díaz et al., 2016; Pugedo et al., 2016). Using this approach, it is possible to identify endemic lineages and shared ichthyofauna. ABGD analyses (Puillandre et al., 2012) were performed using the web interface (^3^web version ‘May 31 2017’) with a relative gap width value of *X* = 1.0 and two available distance metrics [JC69 (Jukes and Cantor, 1969) and K2P (Kimura, 1980)], while the other parameter values employed default settings. The bPTP was conducted using both ML (maximum likelihood) and Bayesian approaches (Zhang et al., 2013). The PTP file input consisted in a nexus tree generated in MrBayes (Ronquist and Huelsenbeck, 2003) using six random parsimony trees, with the GTRGAMMA substitution model (obtained by MEGA 7 under BIC criteria), without rooting and applying the parameters of 20 million MCMC generations and a burn-in of 10%. Analysis was conducted applying default values through the bPTP server (500,000 generations, thinning = 100, burn-in = 10%).

All data, including fish photos, GPS coordinates of each sample site, vouchers numbers, detailed taxonomic identifications, and the corresponding sequence data and trace files were submitted to the BOLD (^2^see Ratnasingham and Hebert, 2007) within the project file ‘DNA Barcoding of DRB’.

### Species Delimitation and Hidden Biodiversity

Species delimitation based on integrative approaches that combine a diverse range of statistical methods has been extensively used to identify hidden biodiversity (i.e., Padial et al., 2010; Costa-Silva et al., 2015; Gomes et al., 2015; Rossini et al., 2016; Ramirez et al., 2017). Here, species with >2% of intraspecific genetic divergences, still undescribed or unknown and identified only at genus or family level were investigated individually to detect the occurrence of new molecular operational taxonomic units (MOTUs) according to the congruence among BIN, ABGD, bPTP outputs.

Undescribed species or those only identified at genus or family level were checked using the BIN and NND analyses in order to verify their occurrence in clusters composed by other nominal species, and their genetic divergence from the nearest neighbor (including species from DRB and/or distinct Brazilian basins). Were considered as new MOTUs when intraspecific genetic divergence was higher than 2% for described species and distinguished clusters identified by BIN, ABGD, and bPTP outputs.

## Results

Morphological identification on the 306 specimens yielded 69 species (see Supplementary Table S1) in of which 43 are native species (five endemic, three threatened by extinction and one endemic and threatened), 13 non-native species and 13 unknown species to the DRB (see Supplementary Table S2), representing over 76% of its known freshwater ichthyofauna (Vieira, 2009). We then obtained 306 partial sequences of the COI gene, consisting of 665 bp on average, and no insertions, deletions, or stop codons were detected, indicating that there was no case of NUMTS (Nuclear mitochondrial DNA sequences) (Song et al., 2008; Hazkani-Covo et al., 2010).

A mean of 4.54 individuals per species were sequenced, comprising 45 genera, 18 families, and 5 orders [Characiformes (41.9%), Siluriformes (40.6%), Perciformes (9.4%), Gymnotiformes (4.7%), and Cyprinodontiformes (3.4%)]. Species represented by one or two specimens (*N* = 19) were not included in the estimation of intraspecific divergences (*Callichthys callichthys*, *Cichla kelberi*, *Clarias gariepinus*, *Hoplosternum littorale*, *Hyphessobrycon bifasciatus*, *H. eques*, *Hypostomus* sp., *Lophiosilurus alexandri*, *Metynnis maculatus*, *Parotocinclus maculicauda*, *Pimelodus maculatus*, *Poecilia vivipara*, *Prochilodus vimboides*, *Pygocentrus nattereri*, *Salminus brasiliensis*, *Steindachneridion doceanum*, *Trichomycterus* aff. *auroguttatus*, *T*. cf. *brasiliensis*, and *T*. *longibarbatus*). The NJ tree identified species-specific clades for 80.9% of all species. The mean genetic distances found within species, genera, and families were: 2.59, 11.4, and 20.5% (**Table 1**), respectively.

**Table 1:** Distance summary reports for sequence divergence between species, genus, and family level, including minimum, mean, and maximum genetic distances (K2P).

	Minimum	Mean	Maximum
	distance (%)	distance (%)	distance (%)
**Within species**	0	2.59	21.82
**Within genera**	0	11.4	24.2
**Within families**	0	20.5	30.99


**FIGURE 2:**
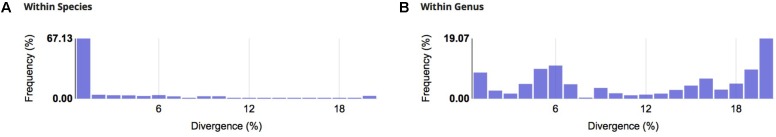
Genetic divergences found for all sequences analyzed at species **(A)** and genus **(B)** levels.

Over 65% of the analyzed species showed genetic distances lower than 1% and for 70% of the species the divergence value was below 2% (**Figure 2A**). When considering intra-generic distance, 19% of the species had a divergence higher than 20% (**Figure 2B**), suggesting the possibility of taxonomic errors or cryptic species.

### Intra- and Inter-Specific Divergence

Intraspecific distance varied from 0 to 21.82%. Particularly high genetic distances (>10%) were recovered among specimens of *Astyanax fasciatus* (20.69%), *Astyanax scabripinnis* (21.82%), *Astyanax* sp. (20.5%), *Characidium* sp. (10.17%), *Crenicichla lacustris* (21.36%), *Harttia* sp. (12.2%), *Poecilia reticulata* (14.34%), and *Trichomycterus* aff. *alternatus* (18.49%), flagging possible new MOTUs (i.e., hidden diversity) or problems related with taxonomic morphological identification.

The NJ tree encompassing all species showed the occurrence of monophyletic clades and absence of shared haplotypes for 44 of the 69 analyzed species. The interspecific genetic distance showed that 63.2% of the analyzed species had a K2P divergence higher than 2% to their closest neighbor, with the exception of: *Astyanax* spp., *Deuterodon pedri*, *H. eques*, *Characidium* sp. and *Characidium* gr. *timbuiense*, *Gymnotus* spp., *Oligosarcus argenteus* and *O. acutirostris*, *P. reticulata* and *P. vivipara,* and *T.* aff. *alternatus* and *T. longibarbatus* (Supplementary Figure S1).

Incongruences between morphological and barcode identifications (BIN, ABGD, bPTP) (i.e., one BIN/ABGD/bPTP cluster containing more than one morphological species, morphological species represented by more than one BIN/ABGD/bPTP cluster, and/or >2% of intraspecific genetic distance and <1% of interspecific divergence) were observed within species of the genus *Astyanax, Characidium*, *Crenicichla, Deuterodon*, *Gymnotus*, *Harttia*, *Hoplias*, *Hyphessobrycon*, *Hypostomus*, *Knodus*, *Neoplecostomus*, *Oligosarcus*, *Pareiorhaphis*, *Poecilia*, *Prochilodus*, *Rhamdia*, and *Trichomycterus* (Supplementary Table S2).

### Identification of Molecular Operational Taxonomic Units (MOTUs)

The BIN analysis identified 81 clusters, including 48 taxonomically concordant, 17 discordant, and 16 singletons. The ABGD analysis detected 54–133 MOTUs when varying the prior maximal distance from *P* = 0.001 to *P* = 0.1000 (applying both the K2P and JC69 nucleotide evolution methods). The partition that recovered 81 groups (intraspecific distance *P* = 0.0077) was chosen due to its consistency with our BIN analysis. The bPTP analyses (Bayesian and ML approaches) resulted in the same number of clusters obtained by BIN, except for *Harttia* sp. (three BIN and ABGD clusters and one bPTP) and *Prochilodus costatus* (two BIN, and one ABGD and bPTP clusters). ABGD species delineation was in agreement with all the BIN clusters with the following exceptions, which contain more than one BIN for each morpho-species: *A. scabripinnis* (BIN: AAC5910, ABGD: 36 and 81), *Knodus moenkhausii* (BIN: AAM1485, ABGD: 46 and 49), *P. costatus* (BIN: ADC2568 and ADC2571, ABGD: 10), *Trichomycterus* sp./*T.* aff. *alternatus*/*T.* aff. *auroguttatus*/*T. longibarbatus* (BIN: ACJ1164 and ACJ1161, ABGD: 64), *Trichomycterus* sp./*T.* cf. *brasiliensis* (BIN: ACK5393 and ACT6325, ABGD: 65) (Supplementary Table S2).

### Identification of Hidden Biodiversity

Sequences from fifteen undescribed species or identified only at genus or family level were compared to other species available in BOLD database through NND and BIN analyses (**Table 2**). Within undescribed or unknown species, we recovered new MOTUs from the following genera: *Astyanax*, *Characidium, Gymnotus, Harttia, Hisonotus, Neoplecostomus, Pareiorhaphis, Phalloceros*, and *Trichomycterus*. The other six species were not considered new MOTUs (*Brycon* sp., *Hasemania* sp., *Hypostomus* sp., *Imparfinis* sp., Neoplecostominae, and *Pimelodella* sp.) since they were included in BINs composed by another nominal species and showed interspecific divergence <2% with the nearest neighbor.

**Table 2:** List of undescribed species, including the nearest neighbor, BIN, and genetic similarity (%).

Species	NND (nearest neighbor species)	BIN	BIN classification	Maximum similarity (%)
*Astyanax* sp.	*Astyanax fasciatus*	AAC5910	Discordant	99.32
	*Deterodon pedri*	ACJ9650	Discordant	99
	*Astyanax intermedius*	ACT0040	Singleton	93.32
	*Astyanax fasciatus, A. bockmanni*	AAY4812	Discordant	99.32
*Brycon* sp.	*Brycon ferox*	ACH8616	Concordant	100
*Characidium sp.*	*Characidium* sp.	ACS9348	Concordant	100
	*Characidium* cf. *timbuiense*	ACJ1226	Discordant	100
	*Characidium* cf. *timbuiense*	ACI3743	Discordant	100
*Gymnotus* sp.	*Gymnotus carapo*	AAB6216	Discordant	100
	*Gymnotus sylvius*	AAB6212	Concordant	100
	*Gymnotus* sp.	ACT0768	Concordant	100
*Harttia* sp.	*Harttia sp.*	ACJ1000	Singleton	100
	*Harttia sp.*	ACI6845	Concordant	100
	*Harttia sp.*	ACO6155	Singleton	100
*Hasemania* sp.	*Hasemania hanseni*	AAO6055	Concordant	100
*Hisonotus* sp.	*Hisotonus* sp.	ACW1732	Concordant	100
*Hypostomus* sp.	*Hypostomus auroguttatus*	AAB9690	Discordant	100
	*Hypostomus heraldoi*			98.32
	*Hypostomus luetkeni*			99.32
	*Hypostomus strigaticeps*			99.32
*Imparfinis* sp.	*Imparfinis minutus*	AAC2103	Concordant	99.32
	*Imparfinis mirini*			98.32
*Neoplecostomus* sp.	*Neoplecostomus* sp.	AAX6581	Concordant	100
	*Neoplecostomus* sp.	ACT2675	Concordant	100
Neoplecostominae	*Pareiohaphis* cf. *bahianus*	ACC0721	Concordant	98.32
*Pareiorhaphis* sp.	*Pareiohaphis scutula*	AAX0824	Discordant	99.32
	*Pareiorhaphis* sp.	ACI5663	Concordant	100
*Phalloceros* sp.	*Phalloceros* sp.	AAB7265	Concordant	100
*Pimelodella* sp.	*Pimelodella lateristriga*	AAC5327	Concordant	99.32
*Trichomycterus* sp.	*Trichomycterus* aff. *immaculatus/T.* cf. *pradensis*	ACI3868	Discordant	99.32
	*Trichomycterus* aff. *auroguttatus*	ACJ1164	Discordant	100
	*Trichomycterus* sp.	ACJ9705	Singleton	98
	*Trichomycterus* cf. *brasiliensis*	ACK5393	Singleton	98.32
	*Trichomycterus* cf. *brasiliensis*	ACT6325	Discordant	99.8


Among species with deep intraspecific divergence (>2%) we recovered additionally at least three putative cryptic species due to the congruence among BIN, ABGD, bPTP, and genetic distance methods for *C. lacustris*, *Hoplias malabaricus*, and *Rhamdia* cf. *quelen* (**Table 3**). *A. fasciatus* and *A. scabripinnis* despite showing a congruence of BIN and ABGD analyses were included in clusters comprising another species of the genus. *K. moenkhausii* had a maximum intraspecific divergence of 3.07% and two distinct ABGD numbers, however, only one clade and one BIN was recovered for this species. *Astyanax lacustris*, *A*. *taeniatus*, *P. reticulata*, *P. costatus*, *T.* aff. *alternatus*, and *T.* aff. *immaculatus* despite showing a high intraspecific genetic distance were included in BINs comprised by another nominal species and thus, were not considered as putative cryptic species.

**Table 3:** List of described species with high intraspecific divergence (>2%), showing the maximum and mean intraspecific genetic distance, clades and number of BIN, ABGD, and bPTP clusters.

Species	Maximum genetic distance (%)	Mean genetic distance (%)	Clades	BIN	ABGD	bPTP
*Astyanax fasciatus*	20.69	10.09	3	3	3	3
*Astyanax lacustris*	3.35	1.67	2	2	2	2
*Astyanax scabripinnis*	21.82	9.12	2	2	3	2
*Astyanax taeniatus*	3.96	1.48	2	2	2	2
*Characidium* sp./*Characidium* cf. *timbuiense^∗^*	10.17/9.9	5.51/5.98	4	4	4	4
*Crenicichla lacustris^∗^*	21.36	10.76	2	2	2	2
*Hoplias malabaricus^∗^*	6.7	3.27	2	2	2	2
*Knodus moenkhausii*	3.07	1.21	1	1	2	1
*Poecilia reticulata*	14.34	9.48	2	2	2	2
*Prochilodus costatus*	2.6	1.32	2	1	1	2
*Rhamdia* cf. *quelen^∗^*	3.48	1.25	2	2	2	2
*Trichomycterus* aff. *alternatus*	18.49	10.8	2	2	2	2
*Trichomycterus* aff. *immaculatus*	5.84	2.23	2	2	2	2


*^∗^Occurrence of cryptic species.*

## Discussion

### DNA Barcoding Effectiveness

We analyzed 306 fish specimens obtained before the dam burst in 2015 and provided genetic data for the ichthyofauna of the DRB, highlighting the occurrence of cryptic and previously unrecognized biodiversity. Therefore, we significantly extend the knowledge on this river system, whose previous surveys mostly focused on the middle course of the river and in lakes located inside the Doce State Park and its surroundings (Sunaga and Verani, 1987; Vieira, 1994; Vono and Barbosa, 2001; Latini and Petrere, 2004). This baseline offers a more robust platform for any future attempt to restore biodiversity and ecosystem functions to a level comparable to pre-disaster conditions.

Using DNA barcoding, we observed an intraspecific genetic distance considerably higher than previously reported for freshwater fish species from other Brazilian basins. On the other hand, intrageneric divergences were found to be similar to previous studies (Carvalho et al., 2011; Pereira et al., 2011; Pugedo et al., 2016). These results suggest a higher occurrence of hidden biodiversity in DRB when compared to other studied Brazilian basins (**Table 4**).

**Table 4:** Comparison among DNA barcoding studies conducted in Brazilian basins, including the number of sequences and species analyzed, and intraspecific and intrageneric distances (minimum and maximum. The mean is inside the parentheses).

Reference	Basin	Number of sequences	Number of species	Intraspecific distance (%)	Intrageneric distance (%)
Pugedo et al., 2016	Jequitinhonha	260	52	0-11.43 (0.44)	1.09-21.55 (12.16)
Nascimento et al., 2016	Itapecuru	440	64	0-8.9 (0.80)	2.65-7.70 (5.13)
Benzaquem et al., 2015^∗^	Amazon	110	14	0-9.8 (2.8)	2.2-22.5 (19.0)
Gomes et al., 2015	Mucuri	141	37	0-3.24 (0.74)	4.29-18.44 (9.5)
Pereira et al., 2013	Upper Paraná	1244	254	0-8.5 (1.3)	0-24.9 (6.8)
Carvalho et al., 2011	São Francisco	431	101	0-10.54 (0.5)	0-22.88 (10.61)
Pereira et al., 2011	Paraíba do Sul	295	58	0-3.48 (0.13)	0.93-22.89 (10.36)
Present study	Doce	306	68	0-21.82 (2.59)	0-24.2 (11.4)


*^∗^Only Nannostomu s spp.*

### Hidden Biodiversity

DNA barcoding has already been used to reveal hidden biodiversity, such as cryptic species and new candidate fish species in the São Francisco (Carvalho et al., 2011), Mucuri (one species – Gomes et al., 2015), and Jequitinhonha (15 species – Pugedo et al., 2016) River catchments. In DRB, from 69 morphologically identified species, the barcode analyses recovered 12 putative cryptic species within *Astyanax* sp., *Characidium* sp., *C.* gr. *timbuiense*, *C. lacustris*, *Gymnotus* sp., *Harttia* sp. (two putative cryptic species), *H. malabaricus*, *Neoplecostomus* sp., *R.* cf. *quelen*, *Trichomycterus* sp. (two putative cryptic species). The high intraspecific genetic distance estimation found for the DRB fish was related to the occurrence of cases of well-known species complexes – e.g., *Astyanax* spp. (maximum intraspecific distance reaching 21.82% in *A. scabripinnis*), *Gymnotus* sp. (6.32%), *H. malabaricus* (6.7%), *R.* cf. *quelen* (3.48%) and also due to the deep intraspecific barcode divergence found to putative overlooked cryptic MOTUs – e.g., *C. lacustris* (21.36%).

DNA barcoding allows for the identification of cryptic variation among morphologically similar species, indicating the occurrence of more than one species and reinforcing the need of an integrative approach combining molecular and morphological characters (Nascimento et al., 2016). By combining distinct species delimitation methods, we were able to identify new MOTUs from nine undescribed species *(Astyanax* sp*., Characidium* sp*., Gymnotus* sp*., Harttia* sp*., Hisonotus* sp*., Neoplecostomus* sp*., Pareiorhaphis* sp.*, Phalloceros* sp., and *Trichomycterus* sp.). Other species showed a high similarity with already described species from another river basins (e.g., specimens of *Brycon* sp. were assigned as *B. ferox* from Mucuri River basin) and were not considered as possible new MOTUs (**Table 2**) as shown by the BIN analysis.

Among the undescribed species, we were able to highlight new MOTUs within five morpho-species due to their high intraspecific genetic divergence and based on BIN, ABGD, and NND analyses. For instance, *Harttia* sp. showed mean divergence of 4.67% and three clades, which were congruent within the BIN and ABGD clustering methods, suggesting the occurrence of three new MOTUs in this genus. Specimens of *Hisonotus* sp. were included in the same BIN/ABGD/bPTP cluster and had an exclusive BIN containing only specimens from DRB suggesting a new MOTU exclusive to this catchment. *Neoplecostomus doceensis* is the only loricariid from this genus described for DRB, however, we found two possible cryptic MOTUs within this taxon, as the DNA barcodes from *Neoplecostomus* sp. did not cluster with barcodes available for this species and had two additional distinct BIN and ABGD clusters. Furthermore, exclusive BIN/ABGD clusters were recovered for *Pareiorhaphis* sp. and *Phalloceros* sp. suggesting at least one new MOTU for each genus endemic to the DRB.

Notwithstanding the high intraspecific genetic distance and species delimitation methods detecting more than one MOTUs, we did not consider new MOTUs for species showing a high similarity with another nominal species (e.g., species comprised in the same BIN cluster as another nominal species). Species with high intraspecific divergence were recovered within *Astyanax* spp. (*A. fasciatus*, *A. lacustris*, *A. scabripinnis*, and *A. taeniatus*). Despite showing a deep intraspecific divergence, and congruence of BIN/ABGD clusters, these species were not considered as comprising new MOTUs due to its high genetic similarity with another nominal species (e.g., *Astyanax parahybae*, *A. vermilion*, *Hyphessobrycon* spp., *Deuterodon* sp.) observed within the BIN and NND analysis, and also, because this highly diverse group is a well-known complex of species in need of more systematic studies (Garutti, 1995; Froese and Paulay, 2010; Eschmeyer, 2015).

High intraspecific divergence was also found for *T.* aff. *alternatus* and *T.* aff. *immaculatus*. These species, despite showing a high intraspecific distance (18.49 and 5.84%, respectively), were included in BINs comprised by another nominal species (e.g., *T. longibarbatus*) indicating it may be a case of morphological misidentification and not the occurrence of new MOTUs. This genus has an extensive geographical range and its morphological identification is complex due to the lack of consistent synapomorphies (Barbosa and Costa, 2003). Therefore, further studies combining an integrative approach focusing in these species are required in order to investigate the occurrence of putative cryptic species.

*Prochilodus costatus* showed a high intraspecific divergence (2.6%) and occurrence of two clusters (NJ and BIN analyses). However, this non-native species was not considered as a putative cryptic species since it was included in BINs comprising another non-native species (e.g., *Prochilodus argenteus*, *P. hartii*). As suggested in previous studies, the incongruence between morphological and molecular identification of *P. costatus* may indicate the occurrence of *Prochilodus* hybrids and not due to new MOTUs (Gomes et al., 2015; Sales et al., 2018).

*Poecilia reticulata* is a species introduced worldwide, occuring in more than 69 countries outside of its native range (Deacon et al., 2011). A high intraspecific divergence (14.34%) was found for this species in the DRB. However, two specimens of *P. reticulata* were assigned to a BIN comprising specimens of *P*. *vivipara* (BIN AAC0279) and the high intraspecific divergence was due to the incongruence between morphological and molecular identification and not due to the occurrence of new MOTUs. Hybridization process between congeneric species of *Poecilia (P. velifera* or *P. petenensis* and *P. mexicana* or *P. orri)* and between different populations of *P. reticulata* have already been reported (Kittell et al., 2005; Lampert and Schartl, 2008; Sievers et al., 2012) and the incongruence detected in this study might be a case of hybridization between *P. reticulata* and *P. vivipara* or misidentification during the deposit in the museum collection and not due to the occurrence of cryptic species.

Hidden biodiversity was found within the genera *Characidium*, *Crenicichla*, *Gymnotus*, *Hoplias*, and *Rhamdia* due to high intraspecific genetic divergence and congruence among clustering methods BIN, ABGD, and bPTP (**Table 3**). Species of the genera *Rhamdia*, *Characidium*, *Pareiorhaphis*, *Gymnotus* were also flagged as cryptic and/or candidate species in other Brazilian basins (Carvalho et al., 2011; Gomes et al., 2015; Pugedo et al., 2016).

For instance, within the genera *Characidium* spp. we detected a mean intraspecific divergence of 5.82% and the occurrence of four clades, of which: two mixed clades comprising specimens identified as *C.* gr. *timbuiense* (*n* = 3 and *n* = 4) and *Characidium* sp. (*n* = 1), one clade exclusive to *C.* gr. *timbuiense* (*n* = 1) and one clade exclusive to *Characidium* sp. (*n* = 4). *C. lacustris* showed intraspecific divergence of 10.76% and presence of two different clades and BIN/ABGD/bPTP clusters (one for samples collected in Manhuaçu River and one for samples collected below the Baguari Dam). The electric knifefishes *Gymnotus* spp. had an intraspecific divergence above 2% and occurrence of three different clades corroborated by 3 BIN, ABGD, and bPTP clusters. All *Gymnotus* specimens were initially morphologically identified as *Gymnotus* sp. and *Gymnotus* cf. *carapo*. However, similarly to the findings obtained for this genus in Mucuri River Basin, these clusters may represent two different known species (*G. carapo* and the overlooked species *Gymnotus sylvius*) and a new MOTU yet to be analyzed and properly described (*Gymnotus* sp.). Two congruent BIN, ABGD, and bPTP clusters were identified for both *H. malabaricus* and *Rhamdia* cf. *quelen* (mean intraspecific divergence of 6.7 and 3.48%, respectively) suggesting the occurrence of cryptic species for each of these taxa. The divergence found in *H. malabaricus* may be due to allopatric speciation resulting from geographical barriers enhanced by its sedentary habitat, since one cluster comprised exclusively specimens from Jose Pedro River and the other was exclusive for specimens from Corrente Grande River. High genetic diversity was already reported for this species in other studied systems (Paraná and Tibagi Rivers) suggesting distinct evolutionary lineages, population structuring or occurrence of cryptic species (Dergam et al., 1998; Blanco et al., 2011; Oliveira et al., 2015).

The increase of available barcodes in BOLD database, including adjacent basins, may contribute to expose endemic cryptic species and reduce the risk of synonymies (Gomes et al., 2015). However, Pugedo et al. (2016) highlighted the concern of using solely DNA barcodes in defining species (e.g., using NND, BIN, ABGD, and bPTP analyses) due to the fact that Neotropical DNA barcode libraries are not yet complete. Furthermore, specimens included in BINs composed by different nominal species should be re-evaluated by a taxonomist to verify the data and check for potential misidentifications (Díaz et al., 2016).

Thus, a thorough analysis should be done for each flagged species to verify the correspondence of new MOTUs with putative new candidate species based on accurate morphological taxonomy analysis and to evaluate the divergence causes and the correlation of speciation process to natural or anthropogenic causes (e.g., presence of dams).

### Importance of DNA Barcoding Library for the Doce River Ichthyofauna

This newly developed DNA barcode reference library for the DRB fish detected the occurrence of new MOTUs and suggested the existence of hidden biodiversity. This baseline information will provide a platform for several applications and management efforts, such as ichthyoplankton identification for the detection of fish recruitment areas, unambiguous choice of species to be used in restocking programs, and environmental DNA research. This data may contribute as a baseline for restoration programs in this catchment, by pointing out new MOTUs and suggesting the occurrence of overlooked and cryptic species among the DRB ichthyofauna, highlighting the complexity of Neotropical biodiversity.

The evidence presented here calls for a more robust, DNA-assisted cataloging of biodiversity-rich ecosystems, in order to enable effective monitoring and informed actions to preserve and restore delicate habitats, such as the DRB. Furthermore, studies should verify the extent to which fish biodiversity has been affected by the Doce dam collapse disaster, and what hotspots of diversity within the catchment can be identified as potential sources of replenishment. At the same time, the approaches used here, and additional high through-put methodologies (e.g., metabarcoding of water and sediment samples) should be increasingly employed to monitor biodiversity at a pace that can cater for the management needs of these increasingly impacted biodiverse habitats.

## Author Contributions

NS performed the molecular genetic analyses and drafted the manuscript. GS and TP collected the samples, conducted the morphological analyses and contributed to the correction of the text. DC designed and coordinates the study. DC and SM conceived the study, participated in its elaboration and helped to draft the manuscript. All authors read and approved the final manuscript.

## Conflict of Interest Statement

The authors declare that the research was conducted in the absence of any commercial or financial relationships that could be construed as a potential conflict of interest.

## Footnotes

1 www.DnaBaser.com

2 http://www.boldsystems.org

3 http://wwwabi.snv.jussieu.fr/public/abgd/abgdweb.html

## ABBREVIATIONS

BIN: barcode index number
BOLD: Barcode of Life Data System
DRB: Doce River Basin
NND: nearest neighbor distance
