# Supplementary material for: Hidden Diversity Hampers Conservation Efforts in a Highly Impacted Neotropical River System
Source: Front Genet. 2018 Jul 24;9:271. doi: 10.3389/fgene.2018.00271 (PMC6066647; doi:10.3389/fgene.2018.00271)
Supplement: Supplementary file 1 [file Image_1.PDF]

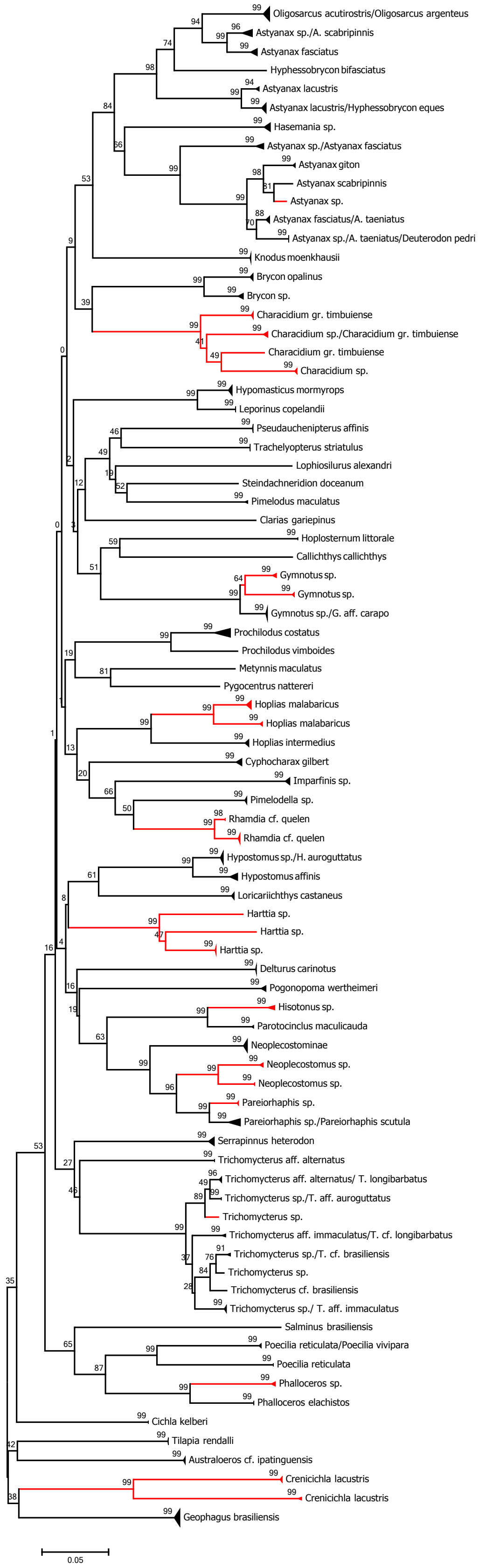

Figure S1: NJ tree based on K2P distance, encompassing all analyzed species to check the occurrence of putative new MOTUs (red branches).
