# Supplementary material for: Hidden Diversity Hampers Conservation Efforts in a Highly Impacted Neotropical River System
Source: Front Genet. 2018 Jul 24;9:271. doi: 10.3389/fgene.2018.00271 (PMC6066647; doi:10.3389/fgene.2018.00271)
Supplement: Supplementary file 2 [file Table_1.DOCX]

Table S1: Sixty-nine fish species barcoded from Doce River Basin and identified as a unique Barcode Index Number (BIN). LGC and RD– Laboratório de Genética da Conservação do Programa de Pós-graduação em Biologia de Vertebrados/PUC Minas, MBML – Museu de Biologia Professor Mello Leitão, MCNIP – Coleção de Ictiologia do Museu de Ciências Naturais da PUC Minas, ZUEC – Museu de Zoologia da Universidade Estadual de Campinas “Prof. Adão José Cardoso”.

|  |  |  |  |  |  |  |
| --- | --- | --- | --- | --- | --- | --- |
|  |  |  |  |  |  |  |
| **Process ID** | **Sample ID** | **Museum ID** | **Identification** | **Lat** | **Lon** | **BIN** |
| RDOCE175-14 | RD116 | RD116 | *Astyanax fasciatus* | -19.996 | -41.739 | BOLD:ABU7523 |
| RDOCE229-14 | LGC3525 | BG-06VI-37 | *Astyanax fasciatus* | -18.996 | -42.225 | BOLD:ACJ1542 |
| RDOCE231-14 | LGC3545 | BG-12XII-06 | *Astyanax fasciatus* | -18.996 | -42.225 | BOLD:ACJ1542 |
| RDOCE234-14 | LGC3688 | LGC3688 | *Astyanax fasciatus* | -19.004 | -43.375 | BOLD:ACJ9650 |
| RDOCE284-14 | LGC3567 | LGC3567 | *Astyanax fasciatus* | -18.996 | -42.225 | BOLD:ACJ1542 |
| RDOCE285-14 | LGC3568 | LGC3568 | *Astyanax fasciatus* | -18.996 | -42.225 | BOLD:ACJ1542 |
| RDOCE286-14 | LGC3569 | LGC3569 | *Astyanax fasciatus* | -18.996 | -42.225 | BOLD:ACJ1542 |
| RDOCE212-14 | LGC4145 | MBML6827 | *Astyanax giton* | -19.837 | -40.555 | BOLD:ACL8007 |
| RDOCE214-14 | LGC4153 | MBML6842 | *Astyanax giton* | -19.889 | -40.576 | BOLD:ACL8007 |
| RDOCE321-15 | LGC4147 | MBML6831 | *Astyanax giton* | -19.884 | -40.575 | BOLD:ACL8007 |
| RDOCE091-13 | LGC1819 | MCNI-PUCMG-0476 | *Astyanax lacustris* | -20.079 | -41.733 | BOLD:ABZ1711 |
| RDOCE018-13 | LGC153 | MCNI-PUCMG-0476 | *Astyanax lacustris* | -20.079 | -41.733 | BOLD:ABZ1711 |
| RDOCE178-14 | RD134 | RD134 | *Astyanax lacustris* | -19.996 | -41.739 | BOLD:ABZ1711 |
| RDOCE179-14 | RD136 | RD136 | *Astyanax lacustris* | -18.937 | -42.045 | BOLD:ABY8634 |
| RDOCE180-14 | RD137 | RD137 | *Astyanax lacustris* | -18.993 | -42.225 | BOLD:ABY8634 |
| RDOCE152-13 | RD139 | RD139 | *Astyanax lacustris* | -19.062 | -42.162 | BOLD:ABY8634 |
| RDOCE221-14 | RD132 | RD132 | *Astyanax lacustris* | -19.973 | -41.725 | BOLD:ABZ1711 |
| RDOCE222-14 | RD133 | RD133 | *Astyanax lacustris* | -19.973 | -41.725 | BOLD:ABZ1711 |
| RDOCE250-14 | LGC4598 | MCNIP-1607 | *Astyanax lacustris* | -19.985 | -41.722 | BOLD:ABZ1711 |
| RDOCE176-14 | RD121 | RD121 | *Astyanax scabripinnis* | -20.04 | -41.93 | BOLD:AAC5910 |
| RDOCE218-14 | RD120 | RD120 | *Astyanax scabripinnis* | -20.024 | -43.460 | BOLD:AAC5910 |
| RDOCE219-14 | RD123 | RD123 | *Astyanax scabripinnis* | -20.110 | -43.400 | BOLD:AAC5910 |
| RDOCE220-14 | RD124 | RD124 | *Astyanax scabripinnis* | -20.110 | -43.400 | BOLD:AAC5910 |
| RDOCE237-14 | LGC3727 | 83 | *Astyanax scabripinnis* | -18.913 | -43.439 | BOLD:ACO5464 |
| RDOCE142-13 | RD68 | RD68 | *Astyanax* sp. | -20.083 | -43.420 | BOLD:ACJ9650 |
| RDOCE143-13 | RD69 | RD69 | *Astyanax* sp. | -20.083 | -43.420 | BOLD:AAY4812 |
| RDOCE144-13 | RD70 | RD70 | *Astyanax* sp. | -20.083 | -43.420 | BOLD:AAC5910 |
| RDOCE272-14 | RD159 | RD159 | *Astyanax* sp. | -19.014 | -43.377 | BOLD:ACT0040 |
| RDOCE287-14 | LGC3695 | LGC3695 | *Astyanax* sp. | -19.011 | -43.372 | BOLD:ACJ9650 |
| RDOCE297-15 | LGC3724 | LGC3724 | *Astyanax* sp. | -19.011 | -43.372 | BOLD:ACJ9650 |
| RDOCE183-14 | RD148 | RD148 | *Astyanax taeniatus* | -18.967 | -42.318 | BOLD:ABU7523 |
| RDOCE184-14 | RD149 | RD149 | *Astyanax taeniatus* | -18.967 | -42.318 | BOLD:ABU7523 |
| RDOCE153-13 | RD140 | RD140 | *Astyanax taeniatus* | -20.120 | -43.400 | BOLD:ABU7523 |
| RDOCE223-14 | RD142 | RD142 | *Astyanax taeniatus* | -20.120 | -43.400 | BOLD:AAY4812 |
| **Process ID** | **Sample ID** | **Museum ID** | **Identification** | **Lat** | **Lon** | **BIN** |
| RDOCE271-14 | RD141 | RD141 | *Astyanax taeniatus* | -20.121 | -43.401 | BOLD:ABU7523 |
| RDOCE203-14 | LGC1533 | LGC1533 | *Australoheros* cf. *ipatinguensis* | -19.275 | -42.425 | BOLD:ACR9799 |
| RDOCE204-14 | LGC1534 | LGC1534 | *Australoheros* cf. *ipatinguensis* | -19.275 | -42.425 | BOLD:ACR9799 |
| RDOCE205-14 | LGC1535 | LGC1535 | *Australoheros* cf. *ipatinguensis* | -19.275 | -42.425 | BOLD:ACR9799 |
| RDOCE206-14 | LGC1536 | LGC1536 | *Australoheros* cf. *ipatinguensis* | -19.275 | -42.425 | BOLD:ACR9799 |
| RDOCE207-14 | LGC1537 | LGC1537 | *Australoheros* cf. *ipatinguensis* | -19.275 | -42.425 | BOLD:ACR9799 |
| RDOCE189-14 | LGC3678 | LGC3678 | *Brycon opalinus* | -19.289 | -43.192 | BOLD:ACL7114 |
| RDOCE199-14 | LGC3747 | LGC3747 | *Brycon opalinus* | -18.763 | -43.459 | BOLD:ACL7114 |
| RDOCE232-14 | LGC3677 | 1 | *Brycon opalinus* | -19.289 | -43.192 | BOLD:ACL7114 |
| RDOCE233-14 | LGC3679 | 3 | *Brycon opalinus* | -19.289 | -43.192 | BOLD:ACL7114 |
| RDOCE238-14 | LGC3745 | 6 | *Brycon opalinus* | -18.969 | -43.438 | BOLD:ACL7114 |
| RDOCE245-14 | LGC4508 | MCNIP-1602 | *Brycon* sp. | -18.994 | -42.226 | BOLD:ACH8616 |
| RDOCE252-14 | LGC4635 | MCNIP-1601 | *Brycon* sp. | -19.156 | -42.231 | BOLD:ACH8616 |
| RDOCE253-14 | LGC4636 | MCNIP-1601 | *Brycon* sp. | -19.156 | -42.231 | BOLD:ACH8616 |
| RDOCE254-14 | LGC4637 | MCNIP-1601 | *Brycon* sp. | -19.156 | -42.231 | BOLD:ACH8616 |
| RDOCE311-15 | LGC5770 | LGC5770 | *Callichthys callichthys* | -18.91 | -43.442 | BOLD:AAB5066 |
| RDOCE123-13 | RD46 | RD46 | *Characidium* cf. *timbuiense* | -19.022 | -42.122 | BOLD:ACJ1226 |
| RDOCE124-13 | RD48 | RD48 | *Characidium* cf. *timbuiense* | -19.022 | -42.122 | BOLD:ACI3743 |
| RDOCE125-13 | RD51 | RD51 | *Characidium* cf. *timbuiense* | -19.022 | -42.122 | BOLD:ACI3743 |
| RDOCE158-14 | RD47 | RD47 | *Characidium* cf. *timbuiense* | -19.996 | -41.739 | BOLD:ACJ1226 |
| RDOCE159-14 | RD49 | RD49 | *Characidium* cf. *timbuiense* | -18.964 | -42.318 | BOLD:ACI3743 |
| RDOCE140-13 | RD45 | RD45 | *Characidium* cf. *timbuiense* | -19.022 | -42.122 | BOLD:ACJ9733 |
| RDOCE141-13 | RD50 | RD50 | *Characidium* cf. *timbuiense* | -19.002 | -42.127 | BOLD:ACI3743 |
| RDOCE190-14 | LGC3683 | LGC3683 | *Characidium* cf. *timbuiense* | -18.974 | -43.372 | BOLD:ACJ1226 |
| RDOCE198-14 | RD161 | RD161 | *Characidium* sp. | -18.754 | -43.447 | BOLD:ACS9348 |
| RDOCE239-14 | LGC4125 | MBML4422 | *Characidium* sp. | -19.788 | -40.663 | BOLD:ACI3743 |
| RDOCE326-15 | LGC5735 | LGC5735 | *Characidium* sp. | -18.974 | -43.371 | BOLD:ACJ1226 |
| RDOCE327-15 | LGC5719 | LGC5719 | *Characidium* sp. | -18.813 | -43.413 | BOLD:ACS9348 |
| RDOCE328-15 | LGC5752 | LGC5752 | *Characidium* sp. | -18.974 | -43.371 | BOLD:ACS9348 |
| RDOCE230-14 | LGC3540 | BG-11XI-05 | *Cichla kelberi* | -18.996 | -42.225 | BOLD:AAO9230 |
| RDOCE258-14 | LGC4697 | 3440 | *Cichla kelberi* | -19.156 | -42.231 | BOLD:AAO9230 |
| RDOCE094-13 | LGC2674 | LGC2674 | *Clarias gariepinus* | -19.022 | -42.122 | BOLD:AAB2256 |
| RDOCE053-13 | LGC3555 | ZUEC 8147 | *Crenicichla lacustris* | -19.022 | -42.122 | BOLD:AAD6380 |
| RDOCE054-13 | LGC3556 | ZUEC 8147 | *Crenicichla lacustris* | -19.022 | -42.122 | BOLD:AAD6380 |
| RDOCE248-14 | LGC4579 | 17 | *Crenicichla lacustris* | -19.979 | -41.714 | BOLD:ACO6050 |
| RDOCE249-14 | LGC4582 | 26 | *Crenicichla lacustris* | -19.979 | -41.714 | BOLD:ACO6050 |
| RDOCE294-14 | LGC4978 | ZUEC 8199 | *Crenicichla lacustris* | -19.979 | -41.714 | BOLD:ACO6050 |
| RDOCE295-14 | LGC4979 | ZUEC 8199 | *Crenicichla lacustris* | -19.979 | -41.714 | BOLD:ACO6050 |
| RDOCE090-13 | LGC1810 | MCNI-PUCMG-0458 | *Cyphocharax gilbert* | -19.988 | -41.72 | BOLD:ACK1539 |
| RDOCE100-13 | LGC3548 | ZUEC 8153 | *Cyphocharax gilbert* | -18.945 | -42.363 | BOLD:ACK1539 |
| RDOCE101-13 | LGC3550 | ZUEC 8153 | *Cyphocharax gilbert* | -18.945 | -42.363 | BOLD:ACK1539 |
| RDOCE016-13 | LGC143 | MCNI-PUCMG-0458 | *Cyphocharax gilbert* | -19.988 | -41.72 | BOLD:ACK1539 |
| RDOCE050-13 | LGC3549 | ZUEC 8153 | *Cyphocharax gilbert* | -18.945 | -42.363 | BOLD:ACK1539 |
| **Process ID** | **Sample ID** | **Museum ID** | **Identification** | **Lat** | **Lon** | **BIN** |
| RDOCE013-13 | LGC129 | MCNI-PUCMG-0444 | *Delturus carinotus* | -20.016 | -41.735 | BOLD:ACC0184 |
| RDOCE014-13 | LGC130 | MCNI-PUCMG-0446 | *Delturus carinotus* | -20.048 | -41.747 | BOLD:ACC0184 |
| RDOCE024-13 | LGC164 | LGC164 | *Delturus carinotus* | -19.986 | -41.716 | BOLD:ACC0184 |
| RDOCE065-13 | LGC163 | LGC163 | *Delturus carinotus* | -19.986 | -41.716 | BOLD:ACC0184 |
| RDOCE130-13 | LGC165 | LGC165 | *Delturus carinotus* | -19.985 | -41.716 | BOLD:ACC0184 |
| RDOCE224-14 | RD58 | RD58 | *Deuterodon pedri* | -19.001 | -42.231 | BOLD:AAY4812 |
| RDOCE273-14 | RD56 | RD56 | *Deuterodon pedri* | -19.002 | -42.231 | BOLD:AAY4812 |
| RDOCE274-14 | RD57 | RD57 | *Deuterodon pedri* | -19.002 | -42.231 | BOLD:AAY4812 |
| RDOCE086-13 | LGC173 | LGC173 | *Geophagus brasiliensis* | -19.988 | -41.72 | BOLD:AAA8514 |
| RDOCE088-13 | LGC179 | LGC179 | *Geophagus brasiliensis* | -19.988 | -41.72 | BOLD:AAA8514 |
| RDOCE110-13 | RD29 | RD29 | *Geophagus brasiliensis* | -19.022 | -42.122 | BOLD:AAA8514 |
| RDOCE111-13 | RD30 | RD30 | *Geophagus brasiliensis* | -19.022 | -42.122 | BOLD:AAA8514 |
| RDOCE112-13 | RD31 | RD31 | *Geophagus brasiliensis* | -19.022 | -42.122 | BOLD:AAA8514 |
| RDOCE113-13 | RD33 | RD33 | *Geophagus brasiliensis* | -19.022 | -42.122 | BOLD:AAA8514 |
| RDOCE114-13 | RD34 | RD34 | *Geophagus brasiliensis* | -19.022 | -42.122 | BOLD:AAA8514 |
| RDOCE021-13 | LGC158 | LGC158 | *Geophagus brasiliensis* | -19.986 | -41.716 | BOLD:AAA8514 |
| RDOCE032-13 | LGC1817 | MCNI-PUCMG-0476 | *Geophagus brasiliensis* | -20.046 | -41.735 | BOLD:AAA8514 |
| RDOCE062-13 | LGC149 | MCNI-PUCMG-0463 | *Geophagus brasiliensis* | -20.046 | -41.735 | BOLD:AAA8514 |
| RDOCE063-13 | LGC159 | LGC159 | *Geophagus brasiliensis* | -19.986 | -41.716 | BOLD:AAA8514 |
| RDOCE154-14 | LGC150 | MCNI-PUCMG-0463 | *Geophagus brasiliensis* | -20.046 | -41.735 | BOLD:AAA8514 |
| RDOCE264-14 | LGC4961 | ZUEC 8208 | *Gymnotus* aff. *carapo* | -19.985 | -41.722 | BOLD:AAB6216 |
| RDOCE279-14 | LGC4618 | LGC4618 | *Gymnotus* aff. *carapo* | -20.046 | -41.735 | BOLD:AAB6216 |
| RDOCE290-14 | LGC4639 | MCNIP-1604 | *Gymnotus* aff. *carapo* | -19.156 | -42.231 | BOLD:AAB6216 |
| RDOCE292-14 | LGC4704 | MCNIP-1608 | *Gymnotus* aff. *carapo* | -19.156 | -42.231 | BOLD:AAB6216 |
| RDOCE310-15 | LGC4914 | ZUEC 8208 | *Gymnotus* aff. *carapo* | -20.046 | -41.735 | BOLD:AAB6216 |
| RDOCE322-15 | LGC4955 | ZUEC 8208 | *Gymnotus* aff. *carapo* | -19.985 | -41.722 | BOLD:AAB6216 |
| RDOCE075-13 | RD08 | RD08 | *Gymnotus* sp. | -20.110 | -43.400 | BOLD:AAB6212 |
| RDOCE076-13 | RD09 | RD09 | *Gymnotus* sp. | -19.996 | -41.739 | BOLD:AAB6216 |
| RDOCE077-13 | RD11 | RD11 | *Gymnotus* sp. | -19.996 | -41.739 | BOLD:AAB6216 |
| RDOCE136-13 | RD10 | RD10 | *Gymnotus* sp. | -19.996 | -41.739 | BOLD:AAB6216 |
| RDOCE301-15 | RD181 | RD181 | *Gymnotus* sp. | -19.996 | -41.739 | BOLD:ACT0768 |
| RDOCE302-15 | RD92 | RD92 | *Gymnotus* sp. | -20.109 | -43.399 | BOLD:AAB6212 |
| RDOCE306-15 | RD12 | RD12 | *Gymnotus* sp. | -20 | -42 | BOLD:ACT0768 |
| RDOCE308-15 | RD180 | RD180 | *Gymnotus* sp. | -18.99 | -42.215 | BOLD:ACT0768 |
| RDOCE309-15 | RD93 | RD93 | *Gymnotus* sp. | -20.109 | -43.399 | BOLD:AAB6212 |
| RDOCE115-13 | RD35 | RD35 | *Harttia* sp. | -19.022 | -42.122 | BOLD:ACJ1000 |
| RDOCE045-13 | LGC3594 | MCNIP-1637 | *Harttia* sp. | -19.023 | -42.125 | BOLD:ACI6845 |
| RDOCE235-14 | LGC3700 | 54 | *Harttia* sp. | -19.011 | -43.372 | BOLD:ACO6155 |
| RDOCE247-14 | LGC4531 | MBML-PEIXES 7783 | *Harttia* sp. | -19.979 | -41.714 | BOLD:ACI6845 |
| RDOCE251-14 | LGC4634 | MCNIP-1606 | *Harttia* sp. | -20.046 | -41.735 | BOLD:ACI6845 |
| RDOCE265-14 | LGC4976 | ZUEC 8218 | *Harttia* sp. | -19.979 | -41.714 | BOLD:ACI6845 |
| RDOCE266-14 | LGC4977 | ZUEC 8218 | *Harttia* sp. | -19.979 | -41.714 | BOLD:ACI6845 |
| RDOCE267-14 | LGC4985 | ZUEC 8218 | *Harttia* sp. | -19.979 | -41.714 | BOLD:ACI6845 |
| **Process ID** | **Sample ID** | **Museum ID** | **Identification** | **Lat** | **Lon** | **BIN** |
| RDOCE071-13 | RD03 | RD03 | *Hasemania* sp. | -20.084 | -43.415 | BOLD:AAO6055 |
| RDOCE072-13 | RD04 | RD04 | *Hasemania* sp. | -20.084 | -43.415 | BOLD:AAO6055 |
| RDOCE073-13 | RD05 | RD05 | *Hasemania* sp. | -20.084 | -43.415 | BOLD:AAO6055 |
| RDOCE074-13 | RD06 | RD06 | *Hasemania* sp. | -20.084 | -43.415 | BOLD:AAO6055 |
| RDOCE236-14 | LGC3703 | 58 | *Hasemania* sp. | -18.933 | -43.447 | BOLD:AAO6055 |
| RDOCE166-14 | RD85 | RD85 | *Hisonotus* sp. | -18.967 | -42.318 | BOLD:ACW1732 |
| RDOCE134-13 | RD01 | RD01 | *Hisonotus* sp. | -19.062 | -42.162 | BOLD:ACW1732 |
| RDOCE323-15 | RD86 | RD86 | *Hisonotus* sp. | -18.952 | -42.36 | BOLD:ACW1732 |
| RDOCE102-13 | LGC3552 | ZUEC 8146 | *Hoplias intermedius* | -19.022 | -42.122 | BOLD:AAB1734 |
| RDOCE051-13 | LGC3551 | ZUEC 8146 | *Hoplias intermedius* | -19.022 | -42.122 | BOLD:AAB1734 |
| RDOCE052-13 | LGC3553 | ZUEC 8146 | *Hoplias intermedius* | -19.022 | -42.122 | BOLD:AAB1734 |
| RDOCE069-13 | LGC3589 | MCNIP-1638 | *Hoplias intermedius* | -20.084 | -43.415 | BOLD:AAB1734 |
| RDOCE097-13 | LGC3532 | ZUEC 8150 | *Hoplias malabaricus* | -18.986 | -42.216 | BOLD:ACI3811 |
| RDOCE098-13 | LGC3533 | ZUEC 8150 | *Hoplias malabaricus* | -18.986 | -42.216 | BOLD:ACI3811 |
| RDOCE099-13 | LGC3541 | MCNIP-1639 | *Hoplias malabaricus* | -18.972 | -42.286 | BOLD:ACI3811 |
| RDOCE022-13 | LGC160 | LGC160 | *Hoplias malabaricus* | -19.986 | -41.716 | BOLD:AAY4779 |
| RDOCE023-13 | LGC162 | LGC162 | *Hoplias malabaricus* | -19.986 | -41.716 | BOLD:AAY4779 |
| RDOCE031-13 | LGC1814 | MCNI-PUCMG-0461 | *Hoplias malabaricus* | -20.046 | -41.735 | BOLD:AAY4779 |
| RDOCE061-13 | LGC147 | MCNI-PUCMG-0461 | *Hoplias malabaricus* | -20.046 | -41.735 | BOLD:AAY4779 |
| RDOCE064-13 | LGC161 | LGC161 | *Hoplias malabaricus* | -19.986 | -41.716 | BOLD:AAY4779 |
| RDOCE068-13 | LGC181 | LGC181 | *Hoplias malabaricus* | -19.988 | -41.720 | BOLD:AAY4779 |
| RDOCE092-13 | LGC1841 | LGC1841 | *Hoplosternum littorale* | -19.988 | -41.72 | BOLD:AAB5068 |
| RDOCE037-13 | LGC1845 | LGC1845 | *Hoplosternum littorale* | -19.988 | -41.72 | BOLD:AAB5068 |
| RDOCE319-15 | LGC4151 | MBML6839 | *Hyphessobrycon bifasciatus* | -19.888 | -40.575 | BOLD:ACT0106 |
| RDOCE241-14 | LGC4139 | MBML6816 | *Hyphessobrycon eques* | -19.47 | -40.184 | BOLD:ABZ1711 |
| RDOCE280-14 | LGC4963 | ZUEC 8198 | *Hypomasticus mormyrops* | -19.979 | -41.714 | BOLD:ACH5050 |
| RDOCE281-14 | LGC4964 | ZUEC 8198 | *Hypomasticus mormyrops* | -19.979 | -41.714 | BOLD:ACH5050 |
| RDOCE282-14 | LGC4965 | ZUEC 8198 | *Hypomasticus mormyrops* | -19.979 | -41.714 | BOLD:ACH5050 |
| RDOCE283-14 | LGC4966 | ZUEC 8198 | *Hypomasticus mormyrops* | -19.979 | -41.714 | BOLD:ACH5050 |
| RDOCE293-14 | LGC4967 | ZUEC 8198 | *Hypomasticus mormyrops* | -19.979 | -41.714 | BOLD:ACH5050 |
| RDOCE296-15 | LGC3715 | LGC3715 | *Hypomasticus mormyrops* | -19 | -43 | BOLD:ACH5050 |
| RDOCE303-15 | LGC3713 | LGC3713 | *Hypomasticus mormyrops* | -19 | -43 | BOLD:ACH5050 |
| RDOCE004-13 | LGC08 | MCNI-PUCMG-0197 | *Hypostomus affinis* | -18.972 | -42.286 | BOLD:AAW9386 |
| RDOCE006-13 | LGC10 | MCNI-PUCMG-0200 | *Hypostomus affinis* | -19.114 | -42.176 | BOLD:AAW9386 |
| RDOCE007-13 | LGC11 | MCNI-PUCMG-0200 | *Hypostomus affinis* | -19.114 | -42.176 | BOLD:AAW9386 |
| RDOCE008-13 | LGC12 | MCNI-PUCMG-0200 | *Hypostomus affinis* | -19.114 | -42.176 | BOLD:AAW9386 |
| RDOCE015-13 | LGC138 | MCNI-PUCMG-0451 | *Hypostomus affinis* | -20.079 | -41.733 | BOLD:AAW9386 |
| RDOCE002-13 | LGC03 | MCNI-PUCMG-0193 | *Hypostomus auroguttatus* | -19.018 | -42.121 | BOLD:AAB9690 |
| RDOCE003-13 | LGC07 | MCNI-PUCMG-0193 | *Hypostomus auroguttatus* | -19.018 | -42.121 | BOLD:AAB9690 |
| RDOCE009-13 | LGC16 | LGC16 | *Hypostomus auroguttatus* | -19.018 | -42.121 | BOLD:AAB9690 |
| RDOCE010-13 | LGC17 | LGC17 | *Hypostomus auroguttatus* | -19.018 | -42.121 | BOLD:AAB9690 |
| RDOCE011-13 | LGC19 | LGC19 | *Hypostomus auroguttatus* | -19.018 | -42.121 | BOLD:AAB9690 |
| RDOCE028-13 | LGC1672 | MCNI-PUCMG-0193 | *Hypostomus auroguttatus* | -19.018 | -42.121 | BOLD:AAB9690 |
| **Process ID** | **Sample ID** | **Museum ID** | **Identification** | **Lat** | **Lon** | **BIN** |
| RDOCE194-14 | LGC3712 | LGC3712 | *Hypostomus* sp. | -18.917 | -43.462 | BOLD:AAB9690 |
| RDOCE313-15 | LGC5786 | LGC5786 | *Hypostomus* sp. | -18.916 | -43.461 | BOLD:AAB9690 |
| RDOCE129-13 | RD55 | RD55 | *Imparfinis* sp. | -19.022 | -42.122 | BOLD:AAC2103 |
| RDOCE080-13 | RD16 | RD16 | *Imparfinis* sp. | -18.967 | -42.318 | BOLD:AAC2103 |
| RDOCE156-14 | RD17 | RD17 | *Imparfinis* sp. | -18.967 | -42.318 | BOLD:AAC2103 |
| RDOCE227-14 | RD76 | RD76 | *Imparfinis* sp. | -19.202 | -42.361 | BOLD:AAC2103 |
| RDOCE276-14 | RD77 | RD77 | *Imparfinis* sp. | -20.111 | -43.4 | BOLD:AAC2103 |
| RDOCE197-14 | RD160 | RD160 | *Knodus moenkhausii* | -18.933 | -43.447 | BOLD:AAM1485 |
| RDOCE215-14 | RD106 | RD106 | *Knodus moenkhausii* | -20.045 | -43.444 | BOLD:AAM1485 |
| RDOCE216-14 | RD107 | RD107 | *Knodus moenkhausii* | -20.045 | -43.444 | BOLD:AAM1485 |
| RDOCE217-14 | RD111 | RD111 | *Knodus moenkhausii* | -19.001 | -42.231 | BOLD:AAM1485 |
| RDOCE268-14 | RD104 | RD104 | *Knodus moenkhausii* | -19.002 | -42.231 | BOLD:AAM1485 |
| RDOCE269-14 | RD110 | RD110 | *Knodus moenkhausii* | -19.002 | -42.231 | BOLD:AAM1485 |
| RDOCE095-13 | LGC3033 | LGC3033 | *Leporinus copelandii* | -19.022 | -42.122 | BOLD:ACI6721 |
| RDOCE030-13 | LGC1811 | MCNI-PUCMG-0459 | *Leporinus copelandii* | -19.978 | -41.714 | BOLD:ACI6721 |
| RDOCE034-13 | LGC1823 | LGC1823 | *Leporinus copelandii* | -19.978 | -41.714 | BOLD:ACI6721 |
| RDOCE155-14 | LGC3544 | BG-12XII-03 | *Leporinus copelandii* | -18.945 | -42.362 | BOLD:ACI6721 |
| RDOCE093-13 | LGC2668 | LGC2668 | *Lophiosilurus alexandri* | -19.022 | -42.122 | BOLD:AAE4855 |
| RDOCE128-13 | RD54 | RD54 | *Loricariichthys castaneus* | -19.022 | -42.122 | BOLD:ACI6497 |
| RDOCE038-13 | LGC2671 | LGC2671 | *Loricariichthys castaneus* | -19.032 | -42.126 | BOLD:ACI6497 |
| RDOCE040-13 | LGC3036 | LGC3036 | *Loricariichthys castaneus* | -19.022 | -42.122 | BOLD:ACI6497 |
| RDOCE208-14 | LGC2672 | LGC2672 | *Loricariichthys castaneus* | -19.032 | -42.126 | BOLD:ACI6497 |
| RDOCE209-14 | LGC3034 | LGC3034 | *Loricariichthys castaneus* | -19.022 | -42.122 | BOLD:ACI6497 |
| RDOCE133-13 | LGC3537 | LGC3537 | *Metynnis maculatus* | -18.985 | -42.216 | BOLD:AAE7443 |
| RDOCE168-14 | RD96 | RD96 | Neoplecostominae | -19.002 | -42.127 | BOLD:ACC0721 |
| RDOCE169-14 | RD97 | RD97 | Neoplecostominae | -19.002 | -42.127 | BOLD:ACC0721 |
| RDOCE170-14 | RD98 | RD98 | Neoplecostominae | -19.002 | -42.127 | BOLD:ACC0721 |
| RDOCE171-14 | RD99 | RD99 | Neoplecostominae | -19.002 | -42.127 | BOLD:ACC0721 |
| RDOCE173-14 | RD103 | RD103 | Neoplecostominae | -19.002 | -42.127 | BOLD:ACC0721 |
| RDOCE148-13 | RD95 | RD95 | Neoplecostominae | -19.022 | -42.125 | BOLD:ACC0721 |
| RDOCE149-13 | RD100 | RD100 | Neoplecostominae | -19.022 | -42.125 | BOLD:ACC0721 |
| RDOCE185-14 | LGC1822 | LGC1822 | Neoplecostominae | -20.079 | -41.733 | BOLD:ACC0721 |
| RDOCE195-14 | LGC3717 | LGC3717 | Neoplecostominae | -18.917 | -43.462 | BOLD:ACC0721 |
| RDOCE172-14 | RD101 | RD101 | *Neoplecostomus* sp. | -20.047 | -41.685 | BOLD:AAX6581 |
| RDOCE150-13 | RD102 | RD102 | *Neoplecostomus* sp. | -20.047 | -41.685 | BOLD:AAX6581 |
| RDOCE192-14 | LGC3699 | LGC3699 | *Neoplecostomus* sp. | -19.011 | -43.372 | BOLD:ACT2675 |
| RDOCE278-14 | LGC3684 | LGC3684 | *Neoplecostomus* sp. | -18.974 | -43.372 | BOLD:ACT2675 |
| RDOCE325-15 | LGC5733 | LGC5733 | *Neoplecostomus* sp. | -18.974 | -43.371 | BOLD:ACT2675 |
| RDOCE103-13 | LGC3554 | ZUEC 8149 | *Oligosarcus acutirostris* | -19.022 | -42.122 | BOLD:AAI3590 |
| RDOCE033-13 | LGC1818 | MCNI-PUCMG-01476 | *Oligosarcus acutirostris* | -19.988 | -41.72 | BOLD:AAI3590 |
| RDOCE048-13 | LGC3546 | LGC3546 | *Oligosarcus acutirostris* | -18.945 | -42.363 | BOLD:AAI3590 |
| RDOCE049-13 | LGC3547 | LGC3547 | *Oligosarcus acutirostris* | -18.945 | -42.363 | BOLD:AAI3590 |
| RDOCE055-13 | LGC3557 | ZUEC 8149 | *Oligosarcus acutirostris* | -19.022 | -42.122 | BOLD:AAI3590 |
| **Process ID** | **Sample ID** | **Museum ID** | **Identification** | **Lat** | **Lon** | **BIN** |
| RDOCE316-15 | LGC4518 | MCNIP-1605 | *Oligosarcus argenteus* | -19.978 | -41.714 | BOLD:AAI3590 |
| RDOCE317-15 | LGC4519 | LGC4519 | *Oligosarcus argenteus* | -19.978 | -41.714 | BOLD:AAI3590 |
| RDOCE318-15 | LGC4516 | LGC4516 | *Oligosarcus argenteus* | -19.978 | -41.714 | BOLD:AAI3590 |
| RDOCE320-15 | LGC3682 | LGC3682 | *Oligosarcus argenteus* | -18.974 | -43.371 | BOLD:AAI3590 |
| RDOCE151-13 | RD138 | RD138 | *Pareiorhaphis scutula* | -20.045 | -43.444 | BOLD:AAX0824 |
| RDOCE188-14 | RD127 | RD127 | *Pareiorhaphis scutula* | -19.275 | -42.425 | BOLD:AAX0824 |
| RDOCE270-14 | RD128 | RD128 | *Pareiorhaphis scutula* | -20.025 | -43.46 | BOLD:AAX0824 |
| RDOCE177-14 | RD129 | RD129 | *Pareiorhaphis* sp. | -19.01 | -43.37 | BOLD:AAX0824 |
| RDOCE131-13 | LGC1850 | LGC1850 | *Pareiorhaphis* sp. | -20.048 | -41.746 | BOLD:ACI5663 |
| RDOCE210-14 | LGC3710 | LGC3710 | *Pareiorhaphis* sp. | -18.917 | -43.462 | BOLD:AAX0824 |
| RDOCE300-15 | RD177 | RD177 | *Pareiorhaphis* sp. | -20 | -42 | BOLD:ACI5663 |
| RDOCE307-15 | RD178 | RD178 | *Pareiorhaphis* sp. | -20 | -42 | BOLD:ACI5663 |
| RDOCE240-14 | LGC4133 | MBML4646 | *Parotocinclus maculicauda* | -19.888 | -40.574 | BOLD:ACO5053 |
| RDOCE242-14 | LGC4148 | MBML6834 | *Parotocinclus maculicauda* | -19.885 | -40.575 | BOLD:ACO5053 |
| RDOCE211-14 | LGC4144 | MBML6826 | *Phalloceros elachistos* | -19.837 | -40.555 | BOLD:ACO4001 |
| RDOCE243-14 | LGC4149 | MBML6835 | *Phalloceros elachistos* | -19.885 | -40.575 | BOLD:ACO4001 |
| RDOCE289-14 | LGC4150 | MBML6837 | *Phalloceros elachistos* | -19.889 | -40.576 | BOLD:ACO4001 |
| RDOCE193-14 | LGC3704 | LGC3704 | *Phalloceros* sp. | -18.933 | -43.447 | BOLD:AAB7265 |
| RDOCE312-15 | LGC5776 | LGC5776 | *Phalloceros* sp. | -18.933 | -43.446 | BOLD:AAB7265 |
| RDOCE314-15 | LGC5788 | LGC5788 | *Phalloceros* sp. | -18.924 | -43.465 | BOLD:AAB7265 |
| RDOCE126-13 | RD52 | RD52 | *Pimelodella* sp. | -19.022 | -42.122 | BOLD:AAC5327 |
| RDOCE127-13 | RD53 | RD53 | *Pimelodella* sp. | -19.022 | -42.122 | BOLD:AAC5327 |
| RDOCE039-13 | LGC3016 | LGC3016 | *Pimelodella* sp. | -19.023 | -42.125 | BOLD:AAC5327 |
| RDOCE132-13 | LGC3015 | LGC3015 | *Pimelodella* sp. | -19.022 | -42.125 | BOLD:AAC5327 |
| RDOCE200-14 | LGC4126 | MBML4423 | *Pimelodella* sp. | -19.788 | -40.663 | BOLD:AAC5327 |
| RDOCE259-14 | LGC4710 | MCNIP-1609 | *Pimelodus maculatus* | -19.081 | -42.159 | BOLD:AAB6504 |
| RDOCE263-14 | LGC4728 | MCNIP-1610 | *Pimelodus maculatus* | -18.951 | -42.361 | BOLD:AAB6504 |
| RDOCE181-14 | RD145 | RD145 | *Poecilia reticulata* | -19.77 | -40.63 | BOLD:AAC0279 |
| RDOCE182-14 | RD146 | RD146 | *Poecilia reticulata* | -19.77 | -40.63 | BOLD:AAC0279 |
| RDOCE138-13 | RD18 | RD18 | *Poecilia reticulata* | -20.042 | -41.698 | BOLD:ACE9037 |
| RDOCE139-13 | RD19 | RD19 | *Poecilia reticulata* | -20.04 | -41.698 | BOLD:ACE9037 |
| RDOCE288-14 | LGC4140 | MBML6817 | *Poecilia vivipara* | -19.47 | -40.184 | BOLD:AAC0279 |
| RDOCE047-13 | LGC3596 | LGC3596 | *Pogonopoma wertheimeri* | -19.023 | -42.125 | BOLD:ACI3792 |
| RDOCE299-15 | LGC4715 | LGC4715 | *Pogonopoma wertheimeri* | -19 | -42 | BOLD:ACI3792 |
| RDOCE304-15 | LGC4701 | LGC4701 | *Pogonopoma wertheimeri* | -19 | -42 | BOLD:ACI3792 |
| RDOCE305-15 | LGC4726 | LGC4726 | *Pogonopoma wertheimeri* | -19 | -42 | BOLD:ACI3792 |
| RDOCE096-13 | LGC3528 | LGC3528 | *Prochilodus costatus* | -19.032 | -42.126 | BOLD:ADK5931 |
| RDOCE056-13 | LGC3559 | LGC3559 | *Prochilodus costatus* | -19.081 | -42.158 | BOLD:ADK5931 |
| RDOCE057-13 | LGC3560 | LGC3560 | *Prochilodus costatus* | -19.081 | -42.158 | BOLD:ADK5931 |
| RDOCE058-13 | LGC3561 | LGC3561 | *Prochilodus costatus* | -19.081 | -42.158 | BOLD:ADK5929 |
| RDOCE059-13 | LGC3562 | LGC3562 | *Prochilodus costatus* | -19.081 | -42.158 | BOLD:ADK5931 |
| RDOCE060-13 | LGC3563 | LGC3563 | *Prochilodus costatus* | -19.081 | -42.158 | BOLD:ADK5929 |
| RDOCE246-14 | LGC4515 | MCNIP-1603 | *Prochilodus vimboides* | -18.951 | -42.361 | BOLD:ACN4578 |
| **Process ID** | **Sample ID** | **Museum ID** | **Identification** | **Lat** | **Lon** | **BIN** |
| RDOCE046-13 | LGC3595 | LGC3595 | *Pseudauchenipterus affinis* | -19.023 | -42.125 | BOLD:AAH8177 |
| RDOCE255-14 | LGC4651 | MCNIP-1598 | *Pseudauchenipterus affinis* | -19.239 | -42.306 | BOLD:AAH8177 |
| RDOCE256-14 | LGC4652 | MCNIP-1598 | *Pseudauchenipterus affinis* | -19.239 | -42.306 | BOLD:AAH8177 |
| RDOCE260-14 | LGC4712 | 3503 | *Pseudauchenipterus affinis* | -19.081 | -42.159 | BOLD:AAH8177 |
| RDOCE261-14 | LGC4713 | 3504 | *Pseudauchenipterus affinis* | -19.081 | -42.159 | BOLD:AAH8177 |
| RDOCE262-14 | LGC4719 | 3525 | *Pseudauchenipterus affinis* | -18.994 | -42.226 | BOLD:AAH8177 |
| RDOCE291-14 | LGC4656 | MCNIP-1599 | *Pygocentrus* *nattereri* | -19.114 | -42.177 | BOLD:ABZ7351 |
| RDOCE105-13 | LGC3572 | LGC3572 | *Rhamdia* cf. *quelen* | -20.106 | -43.403 | BOLD:AAA6322 |
| RDOCE107-13 | LGC3577 | LGC3577 | *Rhamdia* cf. *quelen* | -20.025 | -43.46 | BOLD:AAA6322 |
| RDOCE036-13 | LGC1844 | LGC1844 | *Rhamdia* cf. *quelen* | -19.988 | -41.72 | BOLD:AAA6323 |
| RDOCE041-13 | LGC3578 | LGC3578 | *Rhamdia* cf. *quelen* | -20.025 | -43.46 | BOLD:AAA6322 |
| RDOCE042-13 | LGC3579 | LGC3579 | *Rhamdia* cf. *quelen* | -20.025 | -43.46 | BOLD:AAA6322 |
| RDOCE078-13 | RD13 | RD13 | *Rhamdia* cf. *quelen* | -19.996 | -41.739 | BOLD:AAA6323 |
| RDOCE079-13 | RD15 | RD15 | *Rhamdia* cf. *quelen* | -20.110 | -43.400 | BOLD:AAA6322 |
| RDOCE167-14 | RD91 | RD91 | *Rhamdia* cf. *quelen* | -18.967 | -42.318 | BOLD:AAA6322 |
| RDOCE137-13 | RD14 | RD14 | *Rhamdia* cf. *quelen* | -20.110 | -43.400 | BOLD:AAA6322 |
| RDOCE104-13 | LGC3564 | ZUEC 8145 | *Salminus brasiliensis* | -19.235 | -42.313 | BOLD:AAD2790 |
| RDOCE160-14 | RD60 | RD60 | *Serrapinnus heterodon* | -18.967 | -42.318 | BOLD:AAE1686 |
| RDOCE161-14 | RD61 | RD61 | *Serrapinnus heterodon* | -18.967 | -42.318 | BOLD:AAE1686 |
| RDOCE162-14 | RD62 | RD62 | *Serrapinnus heterodon* | -18.967 | -42.318 | BOLD:AAE1686 |
| RDOCE186-14 | RD59 | RD59 | *Serrapinnus heterodon* | -19.275 | -42.425 | BOLD:AAE1686 |
| RDOCE225-14 | RD63 | RD63 | *Serrapinnus heterodon* | -20.106 | -43.403 | BOLD:AAE1686 |
| RDOCE226-14 | RD65 | RD65 | *Serrapinnus heterodon* | -18.962 | -42.273 | BOLD:AAE1686 |
| RDOCE108-13 | RD27 | RD27 | Tilapia rendalli | -19.022 | -42.122 | BOLD:ABZ6465 |
| RDOCE109-13 | RD28 | RD28 | *Tilapia rendalli* | -19.022 | -42.122 | BOLD:ABZ6465 |
| RDOCE081-13 | RD25 | RD25 | *Tilapia rendalli* | -20.046 | -41.735 | BOLD:ABZ6465 |
| RDOCE082-13 | RD26 | RD26 | *Tilapia rendalli* | -20.046 | -41.735 | BOLD:ABZ6465 |
| RDOCE298-15 | LGC4612 | LGC4612 | *Tilapia rendalli* | -20 | -42 | BOLD:ABZ6465 |
| RDOCE085-13 | LGC170 | LGC170 | *Trachelyopterus striatulus* | -19.986 | -41.716 | BOLD:ACI3769 |
| RDOCE035-13 | LGC1833 | LGC1833 | *Trachelyopterus striatulus* | -19.986 | -41.716 | BOLD:ACI3769 |
| RDOCE043-13 | LGC3588 | LGC3588 | *Trachelyopterus striatulus* | -19.022 | -42.122 | BOLD:ACI3769 |
| RDOCE044-13 | LGC3593 | LGC3593 | *Trachelyopterus striatulus* | -19.032 | -42.126 | BOLD:ACI3769 |
| RDOCE066-13 | LGC169 | LGC169 | *Trachelyopterus* *striatulus* | -19.986 | -41.7164 | BOLD:ACI3769 |
| RDOCE116-13 | RD36 | RD36 | *Trichomycterus* aff. *immaculatus* | -19.022 | -42.122 | BOLD:ACJ1022 |
| RDOCE117-13 | RD39 | RD39 | *Trichomycterus* aff. *immaculatus* | -19.022 | -42.122 | BOLD:ACI3868 |
| RDOCE118-13 | RD40 | RD40 | *Trichomycterus* aff. *immaculatus* | -19.022 | -42.122 | BOLD:ACI3868 |
| RDOCE157-14 | RD37 | RD37 | *Trichomycterus* aff. *immaculatus* | -19.062 | -42.162 | BOLD:ACI3868 |
| RDOCE147-13 | RD94 | RD94 | *Trichomycterus* aff. *immaculatus* | -20.051 | -43.397 | BOLD:ACI3868 |
| RDOCE120-13 | RD42 | RD42 | *Trichomycterus* aff.*alternatus* | -19.022 | -42.122 | BOLD:ACJ1151 |
| RDOCE121-13 | RD43 | RD43 | *Trichomycterus* aff.*alternatus* | -19.022 | -42.122 | BOLD:ACJ1151 |
| RDOCE122-13 | RD44 | RD44 | *Trichomycterus* aff.*alternatus* | -19.022 | -42.122 | BOLD:ACJ1151 |
| RDOCE187-14 | RD88 | RD88 | *Trichomycterus* aff.*alternatus* | -18.953 | -42.361 | BOLD:ACL7294 |
| RDOCE324-15 | RD87 | RD87 | *Trichomycterus* aff.*alternatus* | -18.952 | -42.36 | BOLD:ACL7294 |
| **Process ID** | **Sample ID** | **Museum ID** | **Identification** | **Lat** | **Lon** | **BIN** |
| RDOCE196-14 | RD150 | RD150 | *Trichomycterus* cf. *brasiliensis* | -19.011 | -43.372 | BOLD:ACT6325 |
| RDOCE201-14 | LGC4143 | MBML6825 | *Trichomycterus* *longibarbatus* | -19.837 | -40.555 | BOLD:ACJ1022 |
| RDOCE202-14 | LGC4152 | MBML6841 | *Trichomycterus longibarbatus* | -19.889 | -40.576 | BOLD:ACJ1151 |
| RDOCE163-14 | RD71 | RD71 | *Trichomycterus* sp. | -18.967 | -42.318 | BOLD:ACI3868 |
| RDOCE164-14 | RD72 | RD72 | *Trichomycterus* sp. | -18.967 | -42.318 | BOLD:ACJ1164 |
| RDOCE165-14 | RD73 | RD73 | *Trichomycterus* sp. | -18.967 | -42.318 | BOLD:ACK5393 |
| RDOCE146-13 | RD79 | RD79 | *Trichomycterus* sp. | -20.109 | -43.399 | BOLD:ACJ9705 |
| RDOCE191-14 | LGC3686 | LGC3686 | *Trichomycterus* sp. | -18.974 | -43.372 | BOLD:ACT6325 |
|  |  |  |  |  |  |  |
