# Supplementary material for: Hidden Diversity Hampers Conservation Efforts in a Highly Impacted Neotropical River System
Source: Front Genet. 2018 Jul 24;9:271. doi: 10.3389/fgene.2018.00271 (PMC6066647; doi:10.3389/fgene.2018.00271)
Supplement: Supplementary file 3 [file Table_2.DOCX]

Table S2: Species analyzed from Doce River Basin including intraspecific distance and additional analyses. E=endemic, I=invasive, N=native, T=Threatened, U=Unknown species.

|  |  |  | |  |  |  |  |  |  | |  |  |  | |  |  |
| --- | --- | --- | --- | --- | --- | --- | --- | --- | --- | --- | --- | --- | --- | --- | --- | --- |
| **Species** | **N** | **Comparisons** | | **Intraspecific distance** | | | **Number of MOTUs** | | | | **Number of specimens** | **BIN_ID** | **ABGD_ID** | | **BIN** | **Status** |
|  |  |  |  | **Minimum distance (%)** | **Mean distance (%)** | **Maximum distance (%)** | **BIN** | **ABGD** | **bPTP** | |  |  |  |  |  |  |
| ***Astyanax fasciatus*** | 7 | | 21 | 0 | 10.09 | 20.69 | 3 | 3 | 3 | 1 | | ABU7523 | | 35 | Discordant | N |
|  |  |  |  |  |  |  |  |  |  | 5 | | ACJ1542 | | 76 | Concordant |  |
|  |  |  |  |  |  |  |  |  |  | 1 | | ACJ9650 | | 42 | Discordant |  |
| ***Astyanax giton*** | 3 | | 3 | 0 | 0.52 | 0.84 | 1 | 1 | 1 | 3 | | ACL8007 | | 73 | Concordant | U |
| ***Astyanax lacustris*** | 9 | | 36 | 0 | 1.67 | 3.35 | 2 | 2 | 2 | 3 | | ABY8634 | | 59 | Concordant | N |
|  |  |  |  |  |  |  |  |  |  | 6 | | ABZ1711 | | 5 | Discordant |  |
| ***Astyanax scabripinnis*** | 5 | | 10 | 0 | 9.12 | 21.82 | 2 | 3 | 2 | 4 | | AAC5910 | | 36 | Discordant | N |
|  |  |  |  |  |  |  |  |  |  |  |  |  |  | 81 | Discordant |  |
|  |  |  |  |  |  |  |  |  |  | 1 | | ACO5464 | | 74 | Singleton |  |
| ***Astyanax* sp.** | 6 | | 15 | 0.79 | 8.77 | 20.5 | 4 | 4 | 4 | 1 | | AAC5910 | | 81 | Discordant | N |
|  |  |  |  |  |  |  |  |  |  | 3 | | ACJ9650 | | 42 | Discordant |  |
|  |  |  |  |  |  |  |  |  |  | 1 | | ACT0040 | | 75 | Singleton |  |
|  |  |  |  |  |  |  |  |  |  | 1 | | AAY4812 | | 80 | Discordant |  |
| ***Astyanax taeniatus*** | 5 | | 10 | 0 | 1.48 | 3.96 | 2 | 2 | 2 | 1 | | AAY4812 | | 80 | Discordant | N |
|  |  |  |  |  |  |  |  |  |  | 4 | | ABU7523 | | 35 | Discordant |  |
| ***Australoeros* cf. *ipatinguensis*** | 5 | | 10 | 0 | 0.1 | 0.17 | 1 | 1 | 1 | 5 | | ACR9799 | | 48 | Concordant | N |
| ***Brycon opalinus*** | 5 | | 10 | 0 | 0.22 | 0.47 | 1 | 1 | 1 | 5 | | ACL7114 | | 44 | Concordant | N \| E |
| ***Brycon* sp.** | 4 | | 6 | 0.18 | 0.7 | 1.43 | 1 | 1 | 1 | 4 | | ACH8616 | | 78 | Concordant | N \| T |
| ***Callichthys callichthys*** | 1 | | - | - | - | - | 1 | 1 | 1 | 1 | | AAB5066 | | 56 | Singleton | N \| T |
| ***Characidium* cf. *timbuiense*** | 8 | | 28 | 0 | 5.98 | 9.9 | 3 | 3 | 3 | 4 | | ACI3743 | | 20 | Discordant | N \| T |
|  |  |  |  |  |  |  |  |  |  | 3 | | ACJ1226 | | 19 | Discordant |  |
|  |  |  |  |  |  |  |  |  |  | 1 | | ACJ9733 | | 41 | Singleton |  |
| ***Characidium* sp*.*** | 6 | | 15 | 0 | 5.51 | 10.17 | 3 | 3 | 3 | 4 | | ACS9348 | | 47 | Concordant | U |
|  |  |  |  |  |  |  |  |  |  | 1 | | ACI3743 | | 20 | Discordant |  |
|  |  |  |  |  |  |  |  |  |  | 1 | | ACJ1226 | | 19 | Discordant |  |
| ***Cichla kelberi*** | 1 | | - | - | - | - | 1 | 1 | 1 | 1 | | AAO9230 | | 50 | Concordant | U |
| ***Clarias gariepinus*** | 1 | | - | - | - | - | 1 | 1 | 1 | 1 | | AAB2256 | | 8 | Singleton | I |
| ***Crenicichla lacustris*** | 6 | | 15 | 0 | 10.76 | 21.36 | 2 | 2 | 2 | 2 | | AAD6380 | | 28 | Concordant | I |
|  |  |  |  |  |  |  |  |  |  | 4 | | ACO6050 | | 53 | Concordant |  |
| ***Cyphocharax gilbert*** | 5 | | 10 | 0 | 0.56 | 1.14 | 1 | 1 | 1 | 5 | | ACK1539 | | 4 | Concordant | N |
| ***Delturus carinotus*** | 6 | | 15 | 0 | 0.15 | 0.33 | 1 | 1 | 1 | 6 | | ACC0184 | | 3 | Concordant | N |
| ***Deuterodon pedri*** | 3 | | 3 | 0 | 0.19 | 0.29 | 1 | 1 | 1 | 3 | | AAY4812 | 80 | | Discordant | N \| E |
| **Species** | **N** | | **Comparisons** | **Intraspecific distance** | | | **Number of MOTUs** | | | **Number of specimens** | | **BIN_ID** | **ABGD_ID** | | **BIN** | **Status** |
|  |  |  |  | **Minimum distance (%)** | **Mean distance (%)** | **Maximum distance (%)** | **BIN** | **ABGD** | **bPTP** |  |  |  |  |  |  |  |
| ***Geophagus brasiliensis*** | 12 | | 66 | 0 | 0.39 | 1.22 | 1 | 1 | 1 | 12 | | AAA8514 | 2 | | Concordant | N |
| ***Gymnotus* aff. *carapo*** | 6 | | 15 | 0 | 0.14 | 0.46 | 1 | 1 | 1 | 6 | | AAB6216 | 69 | | Discordant | N |
| ***Gymnotus* sp*.*** | 9 | | 36 | 0 | 3.96 | 6.32 | 3 | 3 | 3 | 3 | | AAB6216 | 69 | | Discordant | U |
|  |  |  |  |  |  |  |  |  |  | 3 | | AAB6212 | 30 | | Concordant |  |
|  |  |  |  |  |  |  |  |  |  | 3 | | ACT0768 | 70 | | Concordant |  |
| ***Harttia* sp*.*** | 8 | | 28 | 0 | 4.67 | 12.2 | 3 | 3 | 1 | 1 | | ACJ1000 | 17 | | Singleton | N |
|  |  |  |  |  |  |  |  |  |  | 6 | | ACI6845 | 25 | | Concordant |  |
|  |  |  |  |  |  |  |  |  |  | 1 | | ACO6155 | 51 | | Singleton |  |
| ***Hasemania* sp.** | 6 | | 15 | 0 | 0.28 | 0.75 | 1 | 1 | 1 | 6 | | AAO6055 | 29 | | Concordant | N |
| ***Hisonotus* sp.** | 3 | | 3 | 0 | 0.55 | 0.83 | 1 | 1 | 1 | 3 | | ACW1732 | 32 | | Concordant | U |
| ***Hoplias intermedius*** | 4 | | 6 | 0 | 0.28 | 0.64 | 1 | 1 | 1 | 4 | | AAB1734 | 12 | | Concordant | N |
| ***Hoplias malabaricus*** | 9 | | 36 | 0 | 3.27 | 6.7 | 2 | 2 | 2 | 6 | | AAY4779 | 61 | | Concordant | N |
|  |  |  |  |  |  |  |  |  |  | 3 | | ACI3811 | 11 | | Concordant |  |
| ***Hoplosternum littorale*** | 2 | | - | - | - | - | 1 | 1 | 1 | 2 | | AAB5068 | 6 | | Concordant | I |
| ***Hyphessobrycon bifasciatus*** | 1 | | - | - | - | - | 1 | 1 | 1 | 1 | | ACT0106 | 57 | | Singleton | U |
| ***Hyphessobrycon eques*** | 1 | | - | - | - | - | 1 | 1 | 1 | 1 | | ABZ1711 | 5 | | Discordant | I |
| ***Hypomasticus mormyrops*** | 7 | | 21 | 0 | 0.13 | 0.49 | 1 | 1 | 1 | 7 | | ACH5050 | 60 | | Concordant | N |
| ***Hypostomus affinis*** | 5 | | 10 | 0 | 0.51 | 1.23 | 1 | 1 | 1 | 5 | | AAW9386 | 68 | | Concordant | N |
| ***Hypostomus auroguttatus*** | 7 | | 21 | 0 | 0.14 | 0.7 | 1 | 1 | 1 | 7 | | AAB9690 | 24 | | Discordant | N |
| ***Hypostomus* sp.** | 2 | | - | - | - | - | 1 | 1 | 1 | 2 | | AAB9690 | 24 | | Discordant | U |
| ***Imparfinis* sp.** | 5 | | 10 | 0 | 0.33 | 0.76 | 1 | 1 | 1 | 5 | | AAC2103 | 23 | | Concordant | N |
| ***Knodus moenkhausii*** | 6 | | 15 | 0 | 1.21 | 3.07 | 1 | 2 | 1 | 1 | | AAM1485 | 46 | | Concordant | N |
|  |  |  |  |  |  |  |  |  |  | 5 | | AAM1485 | 79 | | Concordant |  |
| ***Leporinus copelandii*** | 4 | | 6 | 0 | 0.82 | 2.21 | 1 | 1 | 1 | 4 | | ACI6721 | 9 | | Concordant | N |
| ***Lophiosilurus alexandri*** | 1 | | - | - | - | - | 1 | 1 | 1 | 1 | | AAE4855 | 7 | | Singleton | I |
| ***Loricariichthys castaneus*** | 5 | | 10 | 0.2 | 0.85 | 2.16 | 1 | 1 | 1 | 5 | | ACI6497 | 22 | | Concordant | N |
| ***Metynnis maculatus*** | 1 | | - | - | - | - | 1 | 1 | 1 | 1 | | AAE7443 | 39 | | Singleton | I |
| **Neoplecostominae** | 9 | | 36 | 0 | 0.3 | 0.8 | 1 | 1 | 1 | 9 | | ACC0721 | 33 | | Concordant | U |
| ***Neoplecostomus* sp.** | 5 | | 10 | 0.16 | 3.46 | 6.12 | 2 | 2 | 2 | 2 | | AAX6581 | 34 | | Concordant | N |
|  |  |  |  |  |  |  |  |  |  | 3 | | ACT2675 | 72 | | Concordant |  |
| ***Oligosarcus acutirostris*** | 5 | | 10 | 0 | 0.34 | 0.76 | 1 | 1 | 1 | 5 | | AAI3590 | 13 | | Discordant | U |
| ***Oligosarcus argenteus*** | 5 | | 10 | 0.17 | 0.7 | 1.24 | 1 | 1 | 1 | 5 | | AAI3590 | 13 | | Discordant | N |
| ***Pareiorhaphis scutula*** | 3 | | 3 | 0.16 | 0.46 | 0.75 | 1 | 1 | 1 | 3 | | AAX0824 | 37 | | Discordant | U |
| ***Pareiorhaphis* sp.** | 5 | | 10 | 0 | 2.74 | 4.44 | 2 | 2 | 2 | 2 | | AAX0824 | 37 | | Discordant | N |
|  |  |  |  |  |  |  |  |  |  | 3 | | ACI5663 | 77 | | Concordant |  |

|  |  |  |  |  |  | |  |  |  |  |  |  |  |  |
| --- | --- | --- | --- | --- | --- | --- | --- | --- | --- | --- | --- | --- | --- | --- |
| **Species** | **N** | **Comparisons** | **Intraspecific distance** | | | | **Number of MOTUs** | | | **Number of specimens** | **BIN_ID** | **ABGD_ID** | **BIN** | **Status** |
|  |  |  | **Minimum distance (%)** | **Mean distance (%)** | | **Maximum distance (%)** | **BIN** | **ABGD** | **bPTP** |  |  |  |  |  |
| ***Parotocinclus maculicauda*** | 2 | - | - | - | | - | 1 | 1 | 1 | 2 | ACO5053 | 71 | Concordant | U |
| ***Phalloceros elachistos*** | 3 | 3 | 0 | 0.14 | | 0.43 | 1 | 1 | 1 | 3 | ACO4001 | 49 | Concordant | NE |
| ***Phalloceros* sp.** | 3 | 3 | 0.16 | 0.42 | | 0.72 | 1 | 1 | 1 | 3 | AAB7265 | 45 | Concordant | U |
| ***Pimelodella* sp.** | 5 | 10 | 0 | 0.25 | | 0.48 | 1 | 1 | 1 | 5 | AAC5327 | 21 | Concordant | N |
| ***Pimelodus maculatus*** | 2 | - | - | - | | - | 1 | 1 | 1 | 2 | AAB6504 | 54 | Concordant | I |
| ***Poecilia reticulata*** | 4 | 6 | 0 | 9.48 | | 14.34 | 2 | 2 | 2 | 2 | AAC0279 | 38 | Discordant | I |
|  |  |  |  |  |  |  |  |  |  | 2 | ACE9037 | 40 | Concordant |  |
| ***Poecilia vivipara*** | 1 | - | - | - | | - | 1 | 1 | 1 | 1 | AAC0279 | 38 | Discordant | N |
| ***Pogonopoma wertheimeri*** | 4 | 6 | 0.33 | 0.48 | | 0.78 | 1 | 1 | 1 | 4 | ACI3792 | 27 | Concordant | I |
| ***Prochilodus costatus*** | 6 | 15 | 0 | 1.32 | | 2.6 | 2 | 1 | 1 | 4 | ADC2568 | 10 | Concordant | I |
|  |  |  |  |  |  |  |  |  |  | 2 | ADC2571 | 10 | Concordant |  |
| ***Prochilodus vimboides*** | 1 | - | - | - | | - | 1 | 1 | 1 | 1 | ACN4578 | 52 | Singleton | N \| T |
| ***Pseudauchenipterus affinis*** | 6 | 15 | 0 | 0.52 | | 1.16 | 1 | 1 | 1 | 6 | AAH8177 | 26 | Concordant | N |
| ***Pygocentrus nattereri*** | 1 | - | - | - | | - | 1 | 1 | 1 | 1 | ABZ7351 | 55 | Singleton | I |
| ***Rhamdia* cf. *quelen*** | 9 | 36 | 0 | 1.25 | | 3.48 | 2 | 2 | 2 | 7 | AAA6322 | 15 | Concordant | N |
|  |  |  |  |  |  |  |  |  |  | 2 | AAA6323 | 62 | Concordant |  |
| ***Salminus brasiliensis*** | 1 | - | - | - | | - | 1 | 1 | 1 | 1 | AAD2790 | 14 | Singleton | I |
| ***Serrapinus heterodon*** | 6 | 15 | 0.32 | 0.76 | | 1.36 | 1 | 1 | 1 | 6 | AAE1686 | 31 | Concordant | N |
| ***Steindachneridion doceanum*** | 1 | - | - | - | | - | 1 | 1 | 1 | 1 | ACT0106 | 58 | Singleton | N \| E \|T |
| ***Tilapia rendalli*** | 5 | 10 | 0 | 0 | | 0 | 1 | 1 | 1 | 5 | ABZ6465 | 16 | Concordant | I |
| ***Trachelyopterus striatulus*** | 5 | 10 | 0 | 0.26 | | 1.23 | 1 | 1 | 1 | 5 | ACI3769 | 1 | Concordant | N |
| ***Trichomycterus* aff. *alternatus*** | 5 | 10 | 0 | 10.8 | | 18.49 | 2 | 2 | 2 | 3 | ACJ1161 | 64 | Discordant | N |
|  |  |  |  |  |  |  |  |  |  | 2 | ACL7294 | 43 | Concordant |  |
| ***Trichomycterus* aff. *auroguttatus*** | 1 | - | - | - | | - | 1 | 1 | 1 | 1 | ACJ1164 | 64 | Discordant | U |
| ***Trichomycterus* cf. *brasiliensis*** | 2 | - | - | - | | - | 1 | 1 | 1 | 2 | ACT6325 | 65 | Discordant | N |
| ***Trichomycterus* aff. *immaculatus*** | 5 | 10 | 0.15 | 2.23 | | 5.84 | 2 | 2 | 2 | 4 | ACI3868 | 63 | Discordant | N |
|  |  |  |  |  |  |  |  |  |  | 1 | ACJ1022 | 18 | Discordant |  |
| ***Trichomycterus longibarbatus*** | 2 | - | - | - | | - | 2 | 2 | 2 | 1 | ACJ1022 | 18 | Discordant | U |
|  |  |  |  |  |  |  |  |  |  | 1 | ACJ1161 | 64 | Discordant |  |
| ***Trichomycterus* sp.** | 5 | 10 | 1.54 | 4.17 | | 5.8 | 5 | 5 | 5 | 1 | ACI3868 | 63 | Discordant | N |
|  |  |  |  |  |  |  |  |  |  | 1 | ACJ1164 | 64 | Discordant |  |
|  |  |  |  |  |  |  |  |  |  | 1 | ACT6325 | 67 | Discordant |  |
|  |  |  |  |  |  |  |  |  |  | 1 | ACJ9705 | 66 | Singleton |  |
|  |  |  |  |  |  |  |  |  |  | 1 | ACK5393 | 65 | Singleton |  |
